# Comparison of Empirical Mode Decomposition, Wavelets, and Different Machine Learning Approaches for Patient-Specific Seizure Detection Using Signal-Derived Empirical Dictionary Approach

**DOI:** 10.3389/fdgth.2021.738996

**Published:** 2021-12-13

**Authors:** Muhammad Kaleem, Aziz Guergachi, Sridhar Krishnan

**Affiliations:** ^1^Department of Electrical Engineering, University of Management and Technology, Lahore, Pakistan; ^2^Department of Information Technology Management, Ted Rogers School of Management, Ryerson University, Toronto, ON, Canada; ^3^Department of Electrical, Computer and Biomedical Engineering, Ryerson University, Toronto, ON, Canada

**Keywords:** patient-specific seizure detection, long-term EEG, signal derived dictionary approach, signal decomposition, feature extraction, classification

## Abstract

Analysis of long-term multichannel EEG signals for automatic seizure detection is an active area of research that has seen application of methods from different domains of signal processing and machine learning. The majority of approaches developed in this context consist of extraction of hand-crafted features that are used to train a classifier for eventual seizure detection. Approaches that are data-driven, do not use hand-crafted features, and use small amounts of patients' historical EEG data for classifier training are few in number. The approach presented in this paper falls in the latter category, and is based on a signal-derived empirical dictionary approach, which utilizes empirical mode decomposition (EMD) and discrete wavelet transform (DWT) based dictionaries learned using a framework inspired by traditional methods of dictionary learning. Three features associated with traditional dictionary learning approaches, namely projection coefficients, coefficient vector and reconstruction error, are extracted from both EMD and DWT based dictionaries for automated seizure detection. This is the first time these features have been applied for automatic seizure detection using an empirical dictionary approach. Small amounts of patients' historical multi-channel EEG data are used for classifier training, and multiple classifiers are used for seizure detection using newer data. In addition, the seizure detection results are validated using 5-fold cross-validation to rule out any bias in the results. The CHB-MIT benchmark database containing long-term EEG recordings of pediatric patients is used for validation of the approach, and seizure detection performance comparable to the state-of-the-art is obtained. Seizure detection is performed using five classifiers, thereby allowing a comparison of the dictionary approaches, features extracted, and classifiers used. The best seizure detection performance is obtained using EMD based dictionary and reconstruction error feature and support vector machine classifier, with accuracy, sensitivity and specificity values of 88.2, 90.3, and 88.1%, respectively. Comparison is also made with other recent studies using the same database. The methodology presented in this paper is shown to be computationally efficient and robust for patient-specific automatic seizure detection. A data-driven methodology utilizing a small amount of patients' historical data is hence demonstrated as a practical solution for automatic seizure detection.

## 1. Introduction

Epilepsy is a neurological disorder characterized by seizures caused by sudden abnormalities in the electrical activity of the brain. A large number of people worldwide are affected by epilepsy, which places them at risk of facing effects of seizures such as attention lapses, convulsions and unconsciousness, which may also lead to physical injury (1). Automatic detection of seizures using analysis of electroencephalogram (EEG) signals represents a promising mechanism for diagnosis, long-term monitoring and rehabilitation of epilepsy patients (2). This is however a challenging task due to the non-stationary nature of EEG signals (3). Analysis of multi-channel EEG signals for automatic seizure detection is therefore an active area of research.

For this purpose, analysis of EEG signals and seizure detection using EEG signal recordings has been done using methods from the time, frequency, and time-frequency domains (4). Signal adaptive methods such as empirical mode decomposition and wavelet decomposition have also been extensively used for automatic seizure detection [e.g., (2, 5–10)]. Methods from the domain of dynamic systems, which use, for example, properties of high dimensional phase spaces to capture dynamics of seizures, have also been successful (11). Seizure detection methods have also been classified in terms of linear and non-linear methods (12). Linear methods can be simple as using metrics such as the signal variance or the signal autocorrelation function, or may use time-frequency techniques, such as the discrete wavelet transform. Non-linear methods are based on analysis of non-linear dynamics of EEG signals, and use measures such as the fractal dimension, Lyapunov exponent, or measures derived from information theory such as different forms of entropy (12). A method from the domain of non-linear dynamics in the form of characterization of dynamic behavior of seizures by nullcline analysis in the phase space is presented in Zabihi et al. (13). Discussion of univariate and bivariate measures comprising both linear and non-linear approaches is given in Mormann et al. (14). Methods from the domain of morphological analysis of EEG signals have also found application for seizure detection (12). The common aspect in these approaches from different domains is the hand-crafting or hand-engineering of features, which are then fed into a classifier for automatic seizure detection. Methods from the domain of deep learning are also now being applied to the problem of automatic seizure detection [e.g., (15, 16)].

Dictionary learning approaches represent an important tool for signal and image classification [e.g., (17–21)]. In general, the signal classification problem is formulated in the context of finding a sparse signal representation in a given, over-complete dictionary (22). For classification of test signals, features obtained through sparse decomposition of signals are used, with examples of such features being the reconstruction error and the coefficient vector. There are newer dictionary learning approaches which combine reconstruction and discrimination, such that the over-complete dictionaries are simultaneously reconstructive and discriminative (20). An approach based on a time-frequency dictionary constructed from time and frequency shifts of a parametric function followed by seizure detection based on the reconstruction error is described in Nagaraj et al. (23). Another approach to learn discriminative dictionaries is presented in Akhtar et al. (21), where sparse codes of a test query over the learned dictionary form the input to a classifier.

Over-complete dictionaries of Gabor atoms have been used in conjunction with the matching-pursuit algorithm to extract features for seizure detection. Hand-crafted features, such as the Gabor atom density, normalized Gabor entropy, or regularity statistics based on the Hoelder exponent are used in conjunction with a classifier, such as SVM, for seizure detection (24–27). A method for seizure detection based on atomic decomposition via orthogonal matching pursuit using an overcomplete dictionary of pseudoperiodic Duffing atoms is presented in Nagaraj et al. (22), where the rate of convergence of atomic decomposition is used as a feature for seizure detection.

A signal derived empirical dictionary learning approach for automatic seizure detection has been presented in Kaleem et al. (28). Based on empirical mode decomposition (EMD), this approach, called the EMD-based dictionary approach, is a methodology inspired by traditional methods of dictionary learning. The EMD-based dictionary approach learns a dictionary composed for atoms formed using intrinsic mode functions obtained after decomposing a signal using EMD, and can be used for seizure detection using the projection coefficients as features, obtained after projecting the testing signals against the trained EMD-based dictionary. Seizure detection can be performed using a classifier, whereby a support vector machine classifier was used in Kaleem et al. (28).

Although EMD is a fully data-driven decomposition approach that does not require a basis function, it shares many similarities with the discrete wavelet transform (DWT), such as the decomposition behavior of both techniques corresponding to a dyadic filter-bank (29). In this paper, we extend our previous work in the following significant ways: 1) a DWT-based empirical dictionary approach is introduced, where the atoms of the dictionary are composed of components obtained after decomposition using DWT. 2) For automatic seizure detection, the projection coefficients, coefficient vector and reconstruction error are used as features. These all are features used in traditional dictionary learning techniques, thereby demonstrating the versatility of the signal derived empirical dictionary learning approach. To the best of our knowledge, this is the first time features used in traditional dictionary learning techniques have been successfully applied for automatic seizure detection using empirical EMD and DWT-based dictionaries. 3) The dictionary creation and learning performance of EMD and DWT-based dictionary approaches is compared, and the seizure detection performance of both approaches using all features is also compared. 4) The seizure detection approach is formulated in a realistic scenario, where the classifiers are first trained using small amounts of patients' historical seizure and non-seizure data, and then tested on newer data. The seizure detection results are then also validated using k-fold cross validation, to rule out any bias in the classifier. 5) Seizure detection performance of the methodology is tested with five commonly used classifiers, thereby also enabling a comparison between different classifiers. Additionally, our approach is also distinguished from the other seizure detection approaches from different domains by being data-driven, and not utilizing hand-crafted features, as well as being one of the few studies that utilizes patients' historical data for classifier training.

The methodology proposed in this paper falls in the category of patient-specific seizure detection approaches. For automatic seizure detection, patient-specific approaches are most common [e.g., (7, 13, 28, 30, 31)]. Patient-specific approaches allow more flexible application to different patients, each with their unique EEG patterns. Importantly, a patient-specific seizure detection system can be used for a particular patient as per need, or if newer data for the patient is available. This can result in reduction of the neurologist's burden of monitoring long-term EEG records. Newer patients may also be added independently to the automatic seizure detection system. On the other hand, some studies have employed patient-independent seizure detection as well [e.g., (22)], where the seizure detection results are cross-validated using a leave-one-patient-out scheme that is built on the combined EEG of all patients. Some other studies also describe patient-independent seizure detection approaches [e.g., (32–34)]. However, these approaches randomly take seizure and non-seizure portions of the data for the purpose of seizure detection, and the contribution of such approaches to a real-world scenario is not clear.

## 2. Materials and Methods

### 2.1. EEG Data

This study uses the CHB-MIT scalp EEG database (35), which is a publicly available online database. This database contains multiple long-term EEG recordings from 23 pediatric patients with intractable seizures. These recordings share 23 common channels for each recording, which are, according to the International 10-20 system of electrode positions and nomenclature, FP1-F7, F7-T7, T7-P7, P7-O1, FP1-F3, F3-C3, C3-P3, P3-O1, FP2-F4, F4-C4, C4-P4, P4-O2, FP2-F8, F8-T8, T8-P8, P8-O2, FZ-CZ, CZ-PZ, P7-T7, T7-FT9, FT9-FT10, FT10-T8, and T8-P8. As is the case with previous studies [e.g., (7, 11, 28)], recordings with at least one seizure event are used. The total duration of seizure and non-seizure recordings that have been used is 2.9 and 171 h, respectively.

**Figure 1:**
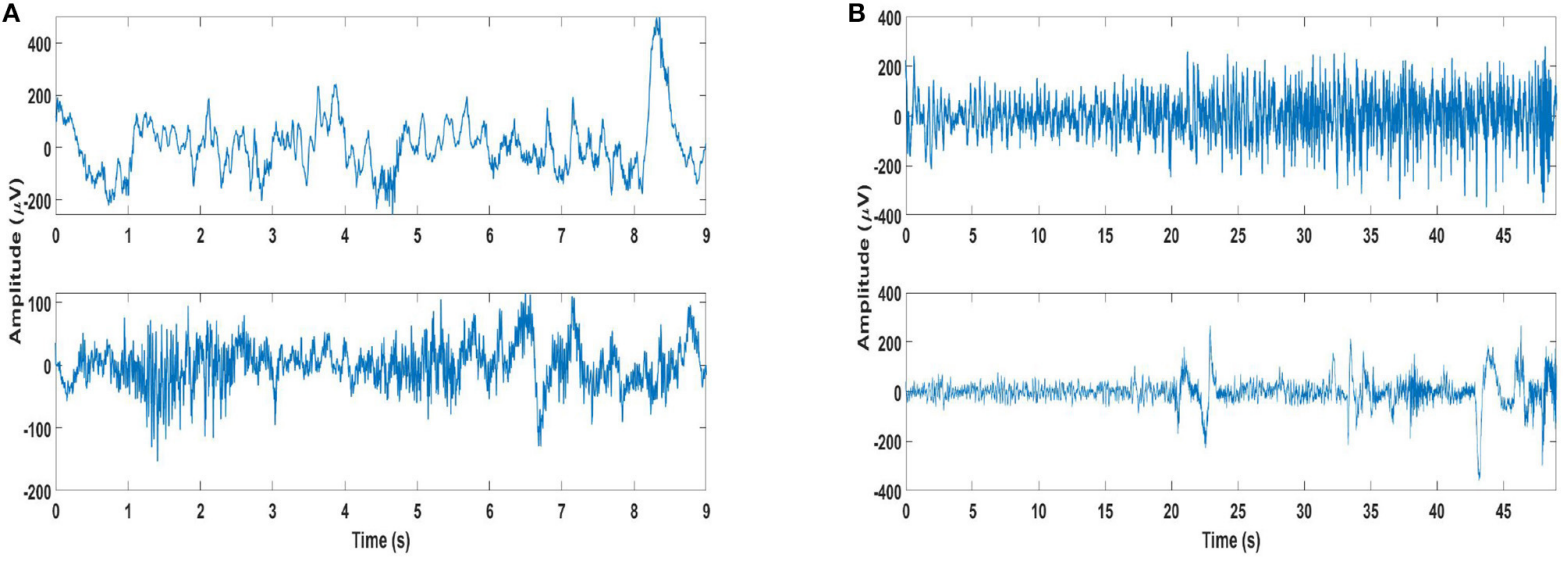
Examples of seizure and non-seizure recordings: **(A)** A 9-s long seizure recording (top plot) of channel 21 of patient 2, and same length of non-seizure signal (bottom plot) from the same recording. **(B)** A 49 s long seizure recording (top plot) of channel 21 of patient 4, and same length of non-seizure signal (bottom plot) from the same recording.

Some important aspects with respect to long-term recordings of scalp EEG may be highlighted as considerable overlap in the seizure and non-seizure EEG, and onset of most seizures accompanied by the development of rhythmic activity consisting of multiple frequency components. Furthermore, the structure of this activity differs from one patient to another, as do the channels on which this activity is most apparent (35). Examples of seizure and non-seizure recordings of two patients from the database are shown in Figure 1, which illustrate these aspects.

Segmentation of the long-term EEG recordings is used for efficient processing, and the recordings are segmented into 4-s lengths for this study. Since the EEG recordings were originally sampled at 256 samples per second, a 4-s segment length contains 1,024 samples. Different lengths have been used in previous studies, with lengths of 1, 2, 3, 4, and even 10 s having been used (2, 7, 11, 33, 36, 37).

One aspect for using shorter segment lengths, such as 1 s, is to ensure stationarity (38), which is not relevant for our approach as our proposed methodology can cater to non-stationary signals as well. Another aspect considered in choosing segment length is associated with resolving the lowest frequency in the EEG signals, which are normally filtered in the range 0.5–60 Hz in the preprocessing stage (39). The proposed methodology does not use any pre-processing, nor is limited to particular frequency bands. In this study we used 4-s long segments based on our previous work using the EMD-based dictionary approach (28), where we had compared 1, 2, 3, and 4 s segments and found 4-s segments to be most suitable. This choice was based on the best results for seizure detection having been obtained using 4 s segments. As our approach does not use hand-crafted features, but instead uses features that capture similarity of testing signals with either the seizure or non-seizure class, longer segments are better able to capture this similarity.

### 2.2. EMD and DWT Decomposition

EMD is a data-adaptive technique that decomposes a signal *x*[*n*] into components called intrinsic mode functions (IMFs) *a*_*j*_[*n*], *j*∈{1, ..., *J*}, such that $x\left[n\right]=\overset{J}{\underset{j=1}{\sum }}a_{j}\left[n\right]$. This decomposition takes place through an iterative process called sifting, which stops after a stopping criterion is fulfilled. Components are considered IMFs if they can be considered zero-mean according to the stopping criterion, and if the number of maxima and minima in the components differs by at most one (40). The EMD algorithm operates at the level of one oscillation, and is adaptive to the local frequency content of the signal. The IMFs demonstrate a dyadic filter-bank behavior (29), though slight deviations from this behavior are possible due to the data-adaptive nature of the decomposition. The IMFs represent a hierarchical separation of a signal's spectral content, with lower index IMFs containing the higher frequency signal components. The number of IMFs *J* is not known in advance, though in general *J* ≤ log_2_(*n*), where *n* is the length of the signal (41). For this work, we used an implementation of the EMD algorithm as presented in Rilling et al. (40).

The DWT can be used to decompose a signal into components, called the details and approximation components, through a pre-defined dyadic sub-band filtering using a basis function called the mother wavelet. These components can be obtained by decomposing a signal *x*[*n*] using DWT given by:


(1) $$\begin{array}{r} x\left[n\right]=\underset{k}{\sum }a_{J,\;k}2^{-J/2}\phi \left(2^{-J}n-k\right)+\underset{j,\;k}{\sum }d_{j,\;k}2^{-j/2}\varphi \left(2^{-j}n-k\right) \end{array}$$

where φ(*n*) is an orthonormal basis function called the mother wavelet, ϕ(*n*) is the scaling function orthogonal to φ(*n*), (*j* = 1, ..., *J*), *k* is the translation parameter, and *d*_*j, k*_ and *a*_*J, k*_ are the details and approximation components, respectively. The number of DWT components depends on the levels specified for decomposition, such that for a *J*-level decomposition there are *J* details and 1 approximation component. In this paper, we utilize the MATLAB implementation of the maximal overlap discrete wavelet transform, which is an energy preserving DWT, with Daubechies db4 as the mother wavelet.

Although the filter-bank structure of IMFs shares similarities with wavelet decomposition, such as in terms of self-similarity, quasi de-correlation and variance progression, wavelet decomposition at each level happens according to a pre-determined frequency division, and the use of linear time-invariant filters for wavelet decomposition does not lend to adaptation to local variations in the frequency content of the signal (42). On the other hand, the data-adaptive nature of EMD lends itself well to non-linear and non-stationary data analysis. In this context, a comprehensive listing of applications of EMD in varied domains is presented in Stallone et al. (43).

### 2.3. Dictionary Learning Algorithm

The signal derived dictionary approach for EMD-based dictionaries has been presented in Kaleem et al. (28). In this section only the main steps of the signal derived dictionary approach are summarized in the context of DWT, and comparison with the EMD-based approach is made where required. We used the db4 mother wavelet for DWT decomposition, which is the most commonly used mother wavelet in seizure detection studies (44), and a 7-level decomposition was used for reasons described later in this section. The background to the selection of the mother wavelet for this study is discussed in further detail later in section 4.1.

The dictionary approach starts with a training matrix ${\text{X}}_{Train}^{c}\in {\mathbb{R}}^{n\;\times \;k^{c}}$. The columns of the matrix consist of *k*^*c*^ training signals *x*^*c*^∈ℝ^*n*^ (1,024 samples length segments of EEG recordings) associated with class *c*. For this work, *c*∈**C**, **C** = {*c*_1_, *c*_2_}, where *c*_1_ represents the seizure class, and *c*_2_ represents the non-seizure class. The first step of the dictionary approach entails forming a raw dictionary ${\text{D}}_{raw}^{\text{C}}={\left\{\psi^{m}\right\}}_{m=1}^{M}$ with *M* atoms ψ, where, in general, *M*<*n*. For the DWT-based dictionary approach, the dictionary atoms ψ are composed of approximation and details components obtained by decomposing signals $x^{c}\in {\text{X}}_{Train}^{c}$ using DWT, whereas for the EMD-based dictionary approach, the dictionary atoms ψ are composed of the IMFs. For DWT, a 7-level decomposition is used in this work, such that 7 details and 1 approximation component are available. For the EMD-based dictionary approach, the average number of IMFs obtained by decomposing all segments, averaged over all patients, was found to be 8. Hence a 7-level DWT decomposition is used, such that the number of components obtained by EMD and DWT decomposition is comparable. Here, we use the notation $a_{q}^{c}\left(n\right),\;q\in \left\{1,...,Q\right\}$ for DWT components as well as the IMFs. The formation of the raw dictionary ${\text{D}}_{raw}^{\text{C}}$ consists of the following sub-steps:

1. For the DWT and EMD-based dictionaries, DWT components and IMFs, respectively, form the atoms of class-specific raw dictionaries ${\text{D}}_{raw}^{c}=\left[{\text{d}}_{1}|{\text{d}}_{2},...,|{\text{d}}_{\text{L}}\right]\in {\mathbb{R}}^{n\times L},\;L=k^{c}\times Q$. The atoms of the class-specific raw dictionaries are constrained to have *l*_2_-norm ≤ 1, so that ${\text{d}}_{l}={\hat{\text{a}}}_{q}^{c},l\in \left\{1,...,L\right\},q\in \left\{1,...,Q\right\}$, and ${\hat{\text{a}}}_{q}^{c}={\text{a}}_{q}^{c}/||{\text{a}}_{q}^{c}|{|}_{2}$. It may be re-iterated that for a 7 level DWT decomposition, *J* = 7, hence *Q* = *J*+1 = 8 whereas *Q* is not known in advance in case of EMD, but for this study, *Q* = 8 on average.
2. A combined raw dictionary
${\text{D}}_{raw}^{\text{C}}=\left[{\text{D}}_{raw}^{c_{1}}|{\text{D}}_{raw}^{c_{2}}\right]\in {\mathbb{R}}^{n\times M}$ is then formed by merging the class-specific raw dictionaries. Here, since **C** = {*c*_1_, *c*_2_}, therefore *M* = 2*L*.

Once the combined raw dictionary is available, a trained dictionary ${\text{D}}_{Train}^{\text{C}}={\left\{\psi^{p}\right\}}_{p=1}^{P}$ is learned from the raw dictionary ${\text{D}}_{raw}^{\text{C}}={\left\{\psi^{m}\right\}}_{m=1}^{M}$. Here, *P* < < *M*, which indicates that the dictionary learning step is accompanied by a significant decrease in dictionary size. The trained dictionary is learned according to a dictionary learning algorithm using the training signals *x*^*c*^, which are the same signals belonging to the seizure and non-seizure classes in the training matrices ${\text{X}}_{Train}^{c_{1}}$ and ${\text{X}}_{Train}^{c_{2}}$. The dictionary learning algorithm is terminated after *I* iterations, where *I* is determined using a validation scheme. The validation scheme uses validation signals ${\hat{x}}^{c}$ belonging to the seizure and non-seizure classes, which are different from the signals *x*^*c*^ used for dictionary creation. The number of validation signals is the same for seizure and non-seizure classes, and is fixed. The trained dictionary ${\text{D}}_{Train}^{\text{C}}$ results from the completion of the dictionary learning algorithm. Based on our initial testing, the steps of the dictionary learning algorithm are repeated up to seven iterations to determine the value of *I* using the validation scheme.

The dictionary learning algorithm consists of the following steps:

1. Initialize an empty trained dictionary ${\text{D}}_{Train}^{\text{C}}=\left[\;\right]$.
2. Initialize a raw dictionary ${\text{D}}_{raw}^{\text{C}}\left(x\right)$ for each training signal *x*.
3. **Repeat** for up to 7 iterations *I*:*I* = 1to7:
  a. For each training signal $x\in {\text{X}}_{Train}^{c_{1}}\cup {\text{X}}_{Train}^{c_{2}}$:
    (1) Compute the projection coefficient α_*m*_ of *x* against each atom ψ^*m*^ in the raw dictionary ${\text{D}}_{raw}^{\text{C}}\left(x\right):\alpha_{m}=<x,\psi^{m}>$;
    (2) Select the atom $\tilde{\psi^{m}}$ whose projection coefficient has the largest absolute value |α_*m*_|;
    (3) **If** $\tilde{\psi^{m}}$ is not in ${\text{D}}_{Train}^{\text{C}}$, **then** add it to the trained dictionary: ${\text{D}}_{Train}^{\text{C}}\leftarrow {\text{D}}_{Train}^{\text{C}}\cup \left\{\tilde{\psi^{m}}\right\}$
    (4) Remove $\tilde{\psi^{m}}$ from the raw dictionary: ${\text{D}}_{raw}^{\text{C}}\left(x\right)\leftarrow {\text{D}}_{raw}^{\text{C}}\left(x\right)\backslash \left\{\tilde{\psi^{m}}\right\}$
    (5) Replace *x* by the residue after projecting on $\tilde{\psi^{m}}:x\leftarrow x-\tilde{\psi^{m}}<x,\tilde{\psi^{m}}>$
b. The trained dictionary ${\text{D}}_{Train}^{\text{C}}$ for the current value of *I* is available.
Then the validation scheme is used:
c. For each class *c*∈{*c*_1_, *c*_2_}:
  (1) Initialize an empty projection coefficients vector Γ_*c*_;
  (2) For each validation signal ${\hat{x}}^{c}$ of class *c*:
    (1) Compute the projection coefficient α_*m*_ of ${\hat{x}}^{c}$ against each atom ψ^*m*^ in the current trained dictionary obtained after *I* iterations ${\text{D}}_{Train}^{\text{C}}:\alpha_{m}=<{\hat{x}}^{c},\psi^{m}>$.
    (2) Select the projection coefficient with the largest absolute value |α_*m*_|, and append this value |α_*m*_| to Γ_*c*_.
d. Calculate the distance between the projection coefficient vectors of both classes: $d=||\Gamma_{c_{1}}-\Gamma_{c_{2}}|{|}_{2}^{2}$.
e. If *d* is the largest distance encountered so far, the current trained dictionary is retained as the best trained dictionary ${\text{D}}_{Train}^{\text{C}}$.
Return the best trained dictionary ${\text{D}}_{Train}^{\text{C}}$.

A few aspects of the dictionary learning algorithm are now mentioned here. The raw dictionary ${\text{D}}_{raw}^{\text{C}}={\left\{\psi^{m}\right\}}_{m=1}^{M}$ has *M* atoms, where *M* = 2*L* = 2(*k*×*Q*), where *k* represents the number of training signals in each training matrix ${\text{X}}_{Train}^{c}$, whereas the trained dictionary ${\text{D}}_{Train}^{\text{C}}={\left\{\psi^{p}\right\}}_{p=1}^{P}$ has *P* atoms. When the same *k* training signals are used for dictionary training, a maximum of 2 × *k* atoms can be added to the trained dictionary in one iteration of dictionary learning. Therefore, the change from raw to trained dictionary is accompanied by a decrease in dictionary size from *M* to *P*. Furthermore, if the number of iterations *I* for dictionary learning is small, then the difference in size between the raw and trained dictionaries is large, such that *P* < < *M*. A smaller dictionary is expected to result in computationally fast feature extraction.

As far as the validation signals ${\hat{x}}^{c}$ are concerned, these are arranged in the order they occur in time in the EEG records. However, the pairing used to calculate the distance between the projection coefficient vectors during the validation stage (Step 3.d) is arbitrary.

### 2.4. Application of Dictionary Learning Approach to the CHB-MIT Database

The signal derived dictionary approach described in the last section is applied to the segmented CHB-MIT database EEG recordings for dictionary creation and learning as follows. Firstly, from each of the 23 channels of each patient, 15% of the seizure segments are used for dictionary creation and learning, and 5% for validation. The same number of segments are selected from non-seizure segments. In addition, 30% of seizure segments are kept for classifier training, with the same number being kept from non-seizure segments. These segments are not selected randomly, instead the order in time is maintained. This means that starting from the beginning of the EEG records, the first 20% segments are kept for dictionary creation, learning and validation, and the next 30% segments for classifier training. The remaining 50% of the seizure segments, and all the remaining non-seizure segments are then kept for testing seizure detection performance. This partitioning of EEG records is meant to mimic a real-world situation, where the system would be trained on existing data, and then used for automatic seizure detection for newly available data (13). The division of available data as mentioned here allows us to keep the later 50% of the EEG records for testing seizure detection, and the earlier 50% for the dictionary approach and classifier training. We chose 20% of the data for dictionary creation, learning and validation, and 30% for classifier training in order to demonstrate the efficacy of our approach using smaller dictionaries, and to have classifier training data comparable to other approaches such as Zabihi et al. (13). For dictionary creation and validation, the 4-s segments obtained from the records of all 23 channels are combined. This allows us to incorporate the multivariate aspect of the data in the dictionary creation and learning approach. The size of the raw dictionary as well as the trained dictionary will be larger when the segments of all 23 channels are combined, compared to the case where dictionaries are created and trained per channel, as in our previous work (28). The trained dictionary is expected to contain the relevant atoms obtained from all the channels after the dictionary learning and validation steps are complete. The total number of segments for each patient for the tasks of dictionary creation, learning and validation, classifier training and testing seizure detection are shown in Supplementary Table S4.

For each patient, a raw dictionary ${\text{D}}_{raw}^{\text{C}}$ is created using the allocated segments combined from all channels. Each raw dictionary is then trained using the dictionary learning steps to obtain ${\text{D}}_{Train}^{\text{C}}$ after *I* iterations, where the number *I* is determined using the validation step, using the segments allocated for validation (having been combined from all channels).

**Figure 2:**
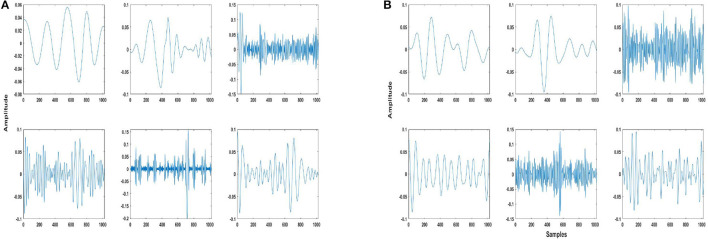
Example of some trained dictionary atoms from **(A)** EMD and **(B)** DWT-based dictionaries of patient 3. These atoms are of 4 s length, or 1,024 samples. The atoms consist of intrinsic mode functions (IMFs)/details/approximation components of both seizure and non-seizure segments, and have been normalized as mentioned in Step 2 of the dictionary approach (section 2.3).

Some examples of trained dictionary atoms for both EMD and DWT-based dictionaries are shown in Figures 2A,B, which illustrate the variety of atoms in the trained dictionary. As already explained previously, these atoms consist of IMFs or DWT details and approximation components. Here we would like to point out that depending on the nature of the application, physical meaning can also be assigned to EMD and DWT components, in the context of understanding different contributions to a given phenomenon made evident through decomposition (43). In this work, however, assigning a physical meaning to the components is not important, as the components form atoms of a dictionary which is trained according to a dictionary learning algorithm, resulting in the selection of atoms most similar to the two classes (seizure and non-seizure). This also precludes requirement of any hand-crafted scheme for selection of IMFs, whereby the important or relevant IMFs have to be selected for a particular application, e.g., seizure detection (45).

A common aspect of the majority of seizure detection studies is pre-processing of EEG records for removal of artifacts [e.g., as mentioned in Mehla et al. (33) and Zabihi et al. (13) etc.]. The dictionary learning algorithm selects atoms (DWT components or IMFs) most similar to the seizure and non-seizure classes for inclusion in the trained dictionary, thereby excluding any components that may contain artifacts. For this reason there is no need for any pre-processing in the proposed methodology, which represents another strength of the proposed approach.

### 2.5. Feature Extraction

In this work, three features are extracted using the trained dictionary, and are listed below. To compare seizure detection performance using these three features, all features are extracted using both EMD and DWT-based dictionaries.

#### 2.5.1. Feature 1: Projection Coefficients (F1)

The projection coefficients are obtained as in dictionary learning Step 3 in section 2.3. The projection coefficient $\alpha_{m}=<x^{c},\psi^{m}>$ having the maximum value is selected as a feature point, where *x*^*c*^ represents all seizure and non-seizure classifier training and testing segments, and ψ^*m*^ represents all atoms of the trained dictionary. The projection coefficients have been used as features in the EMD-based dictionary approach previously (28).

#### 2.5.2. Feature 2: Coefficient Vector (F2)

The coefficients vector **a** is used as a feature vector obtained using the trained dictionary ${\text{D}}_{Train}^{\text{C}}$ and a signal **x**. This feature is obtained using the relation


(2) $$a={\left(D\right)}^{†}x$$

where **x** represents a classifier training or testing signal, and **(*D*)**^†^ is the left pseudo-inverse of the trained dictionary ${\text{D}}_{Train}^{\text{C}}$. Also, since ${\text{D}}_{Train}^{\text{C}}\in {\mathbb{R}}^{n\times P}$, the coefficient vector **a**∈ℝ^*P*^, and hence is a feature vector.

#### 2.5.3. Feature 3: Reconstruction Error (F3)

The reconstruction error ϵ for a classifier training or testing signal **x** is given by ||**x**−**Da**||_2_, where **D** is the trained dictionary and **a** is the coefficient vector. Even though the learned dictionary **D** is not designed as a reconstructive dictionary, it is possible to obtain a coarse reconstruction of the training or testing signal, given by $\hat{\text{x}}$, using the relation


(3) $$\hat{x}=D{\left(D\right)}^{†}x$$

The reconstruction error feature is then obtained as $\epsilon =||\text{x}-\hat{\text{x}}|{|}_{2}$.

#### 2.5.4. All Three Features Combined

In addition to using the three features mentioned above individually, we also use combinations of the three features, and evaluate the seizure detection performance using these combinations. The combinations of the three features F1, F2, and F3 are as follows: all three combined (F1, F2, and F3), Feature 1 and Feature 2 combined (F1 and 2), Feature 1 and Feature 3 combined (F1 and F3) and Feature 2 and Feature 3 combined (F2 and F3).

### 2.6. Classification Methodology

For classification of seizure and non-seizure segments using the three features extracted from the data, five classifiers are used. These are the linear discriminant analysis (LDA) classifier, a support vector machine (SVM) classifier with a radial basis function kernel, the naive-Bayes (NB) classifier, the *k*-nearest neighbor (k-NN) classifier (*k*=1), and classification trees (CT) classifier. Using multiple classifiers allows us to validate our approach with a diverse set of classifiers, as has been done in previous works also [e.g., (7, 46, 47)].

The three features are used to classify seizure and non-seizure segments for each of the 23 EEG channels individually. This is different from the dictionary creation and learning stage, where the seizure and non-seizure segments from all channels are combined. At the seizure detection stage, we use each channel individually as seizures may manifest themselves at different brain locations (13), and hence using each channel individually allows us to better capture the seizures.

Once the classifiers have been trained using the features obtained from seizure and non-seizure segments kept for classifier training, the features extracted from the seizure and non-seizure segments kept for testing are used to evaluate and compare the seizure detection performance of all features and classifiers.

The seizure detection results obtained from each channel are processed in the following simple manner. The area under the receiver operating curve (AUC) is used as a performance measure such that the channel with the highest value of the AUC is selected for seizure detection. Due to this approach, only one channel per patient is used for seizure detection. No channels have to be selected before the seizure detection step [e.g., as in Bhattacharyya and Pachori (7)], nor is any post-processing required, e.g., fusing the results of multiple channels incorporating multiple filters and artificial neural network [e.g., as in Zabihi et al. (11, 13)].

Since we use all channels for seizure detection, the possibility of using the results of more than one channel for seizure detection exists. For this purpose, an extension of the technique described in the last paragraph is used. Instead of using only the channel with the highest value of the AUC, the first three channels ranked in terms of the highest value of the AUC are identified. Then a testing segment is identified as a seizure segment if this segment has been classified as a seizure segment in at least two channels. This technique is applied to patients with seizure detection results lower than a set threshold, and adopted if an improvement in seizure detection results is obtained.

The seizure detection results are evaluated in terms of the performance measures of accuracy, sensitivity and specificity, which are defined in Equation (4). The number of seizure segments correctly classified are represented by *TP* (True Positive), whereas *FN* (False Negative) represents the number of incorrectly classified seizure segments. Non-seizure segments which are correctly classified are given by *TN* (True Negative), and incorrectly classified non-seizure segments are given by *FP* (False Positive).


(4) $$\begin{array}{r} \begin{array}{cc} Sensitivity & =\frac{TP}{TP+FN}\\ Specificity & =\frac{TN}{TN+FP}\\ Accuracy & =\frac{TP+TN}{TP+TN+FP+FN} \end{array} \end{array}$$

## 3. Results

### 3.1. EMD and DWT-based Dictionary Approach Performance

The number of iterations required for dictionary learning termination, averaged over all patients, are 3.26 ± 1.63 and 3.09 ± 1.56 for EMD and DWT-based dictionary approaches, respectively. The DWT-based dictionary is trained using a slightly lesser number of iterations than the EMD-based dictionary. On average, therefore, the size of the DWT-based trained dictionaries is less than those of EMD-based dictionaries, as can be observed in Table 1. The decrease in size going from raw dictionary to trained dictionary is similar on average for both EMD and DWT-based dictionaries, at 68 and 66%, respectively, with similar dispersion around the average. To test the equality of medians of EMD and DWT-based trained dictionaries, the non-parametric Wilcoxon ranksum test was used, which resulted in failure to reject the hypothesis of equal medians.

**Table 1:** Number of atoms in raw and trained dictionaries (variables *M* and *P*, respectively, mentioned in section 2.3) for all subjects for both EMD and DWT-based dictionaries.

**Patient**	**DWT**				**EMD**			
**No**.	** ${\text{D}}_{raw}^{\text{C}}$ **	** ${\text{D}}_{Train}^{\text{C}}$ **	**Iterations I**	**Change (%)**	** ${\text{D}}_{raw}^{\text{C}}$ **	** ${\text{D}}_{Train}^{\text{C}}$ **	**Iterations I**	**Change (%)**
1	5,888	675	1	88	6,018	1,184	2	80
2	2,208	1,192	5	46	2,307	428	2	81
3	5,520	1,258	2	77	5,850	2,826	5	52
4	5,152	2,859	5	44	5,622	2,733	5	51
5	7,728	2,617	3	66	8,040	4,015	5	50
6	1,840	1,028	5	44	1,875	979	5	48
7	4,416	1,959	4	56	4,583	1,822	4	60
8	12,512	1,403	1	89	12,727	2,518	2	80
9	3,680	412	1	89	4,044	387	1	90
10	6,256	2,774	4	56	6,303	3,256	5	48
11	11,040	1,253	1	89	11,727	1,125	1	90
12	13,616	4,620	3	66	13,418	7,230	5	46
13	5,888	2,633	4	55	6,223	1,843	3	70
14	2,208	495	2	78	2,239	637	3	71
15	27,600	9,544	3	65	28,191	14,728	5	48
16	1,104	123	1	89	1,171	216	2	81
17	4,048	1,789	4	56	4,346	2,014	5	54
18	4,416	1,489	3	66	4,520	2,171	5	52
19	3,312	1,840	5	44	3,493	342	1	90
20	4,048	2,248	5	44	4,147	854	2	79
21	2,576	290	1	89	2,729	236	1	91
22	2,944	1,602	5	46	3,187	1,174	4	63
23	5,888	1,992	3	66	6,078	1,235	2	80
Average	6,256	2,004	3.09 ± 1.56	66 ± 17%	6471	2346	3.26 ± 1.63	68 ± 17%

*The number of iterations I required for termination of dictionary learning algorithm is also mentioned (section 2.3)*.

The 7-level DWT decomposition used results in 8 components for each decomposed segment, whereas the number of IMFs resulting from EMD decomposition is not known beforehand. Although the number of IMFs averaged over all segments of all subjects comes to 8, the number of IMFs vary for each 4 s segment, ranging in general between 7 and 10 IMFs. Due to this reason, the number of atoms in the EMD raw dictionaries is in general greater than DWT raw dictionaries.

At the individual patient level, the decrease in dictionary size is comparable for EMD and DWT-based dictionaries. Individual dictionaries that have been trained using a lesser number of iterations have the most decrease in size going from raw to trained dictionary.

As far as decomposition of the signals is concerned, EMD uses an iterative decomposition algorithm that does not require any basis function. On the other hand, selection of a basis function in the form of a mother wavelet is an important aspect of DWT decomposition. This is discussed further in section 4.1.

### 3.2. Classification Performance

**Figure 3:**
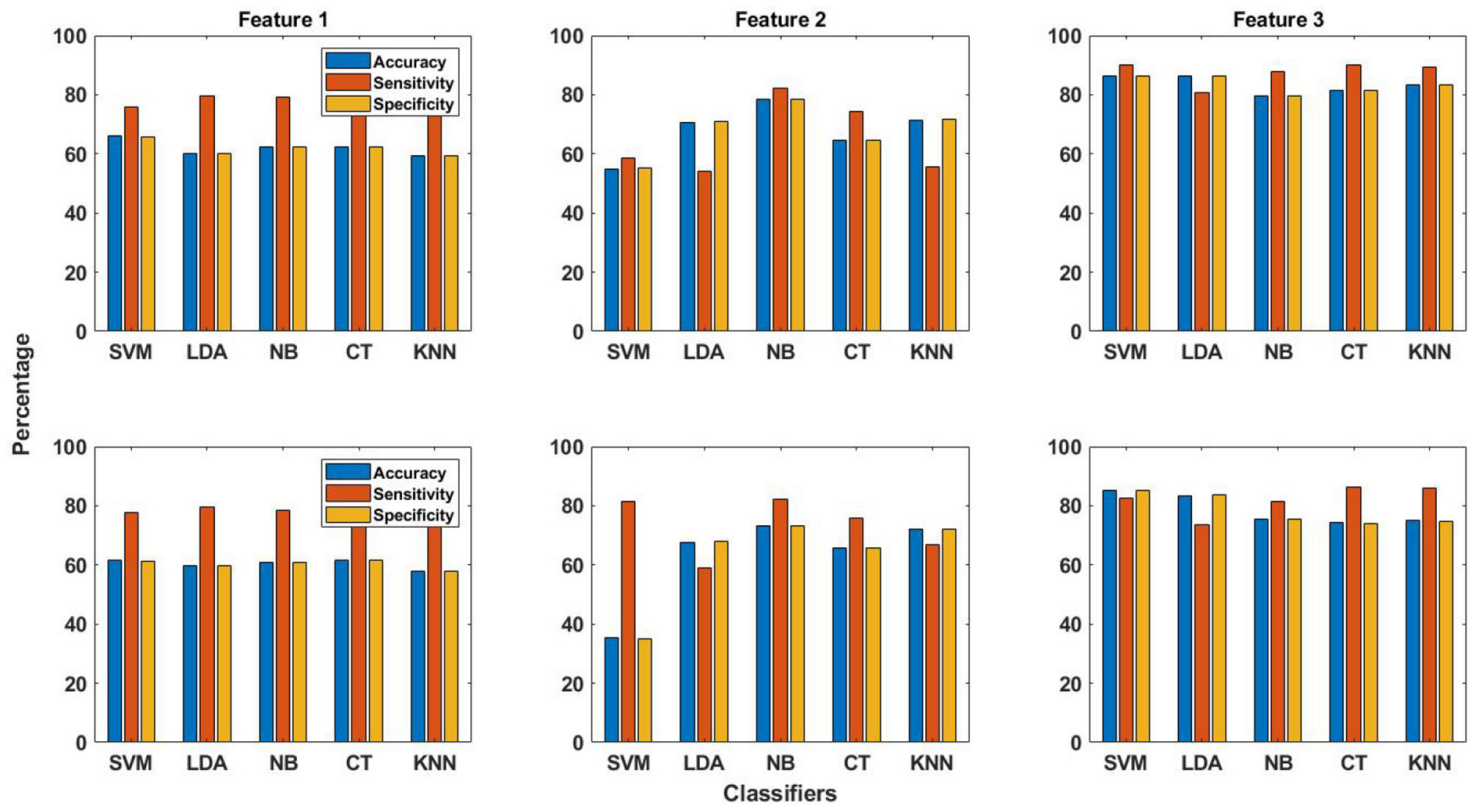
The averaged performance measures of accuracy, sensitivity and specificity obtained using all three features and five classifiers for EMD (top row in figure) and DWT (bottom row in figure) based dictionaries.

The patient-specific seizure detection results in terms of these performance measures were obtained using all features and the five classifiers. For this purpose, the features obtained from the training data are used for training the classifier, whereas classification is performed using the features obtained from the testing data. As already explained in section 2.4, the same number of seizure and non-seizure segments, having occurred earlier in time, are used for obtaining features for training the classifier. This is unlike the work in Zabihi et al. (13), where random under-sampling technique is selected to balance the training data after comparing the performance of different sampling techniques. The seizure detection results in terms of the performance measures for the EMD and DWT-based dictionary approaches are shown in terms of bar graphs in Figure 3. These results have been calculated by selecting the channel with the highest value of AUC (section 2.6). From this figure, it can be observed that on average, the seizure detection performance follows the same pattern for both EMD and DWT-based approaches over all features and classifiers. However, it is clear that the values of accuracy, sensitivity and specificity are higher for EMD-based dictionary approach. In terms of sensitivity, which represents the ability to correctly detect seizures, and hence is of utmost importance (13), Feature 3 (reconstruction error ϵ) performs best for seizure detection across all classifiers, for both EMD and DWT-based approaches. Patient-specific seizure detection results in terms of accuracy, sensitivity and specificity using Features 1, 2 and 3, for all 5 classifiers and both EMD and DWT-based dictionaries are shown in Supplementary Tables S1–S3.

**Figure 4:**
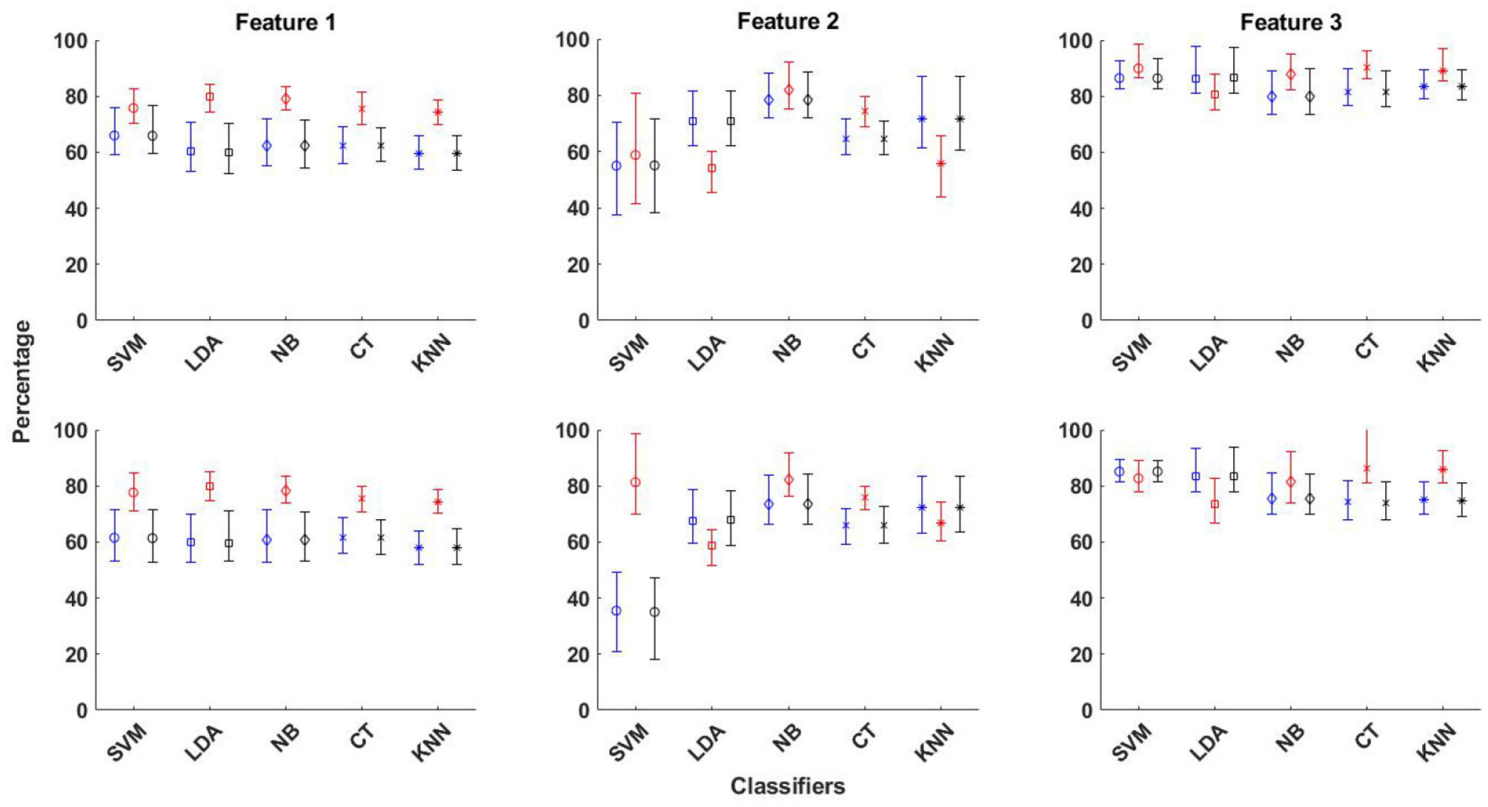
The averaged performance measures of accuracy, sensitivity and specificity obtained using all three features and five classifiers for EMD (top row in figure) and DWT (bottom row in figure) based dictionaries, shown with upper and lower confidence bounds for the 95% confidence intervals.

The best averaged sensitivity value is obtained for Feature 3 using SVM classifier (89.9%) and EMD-based dictionary. This is followed by the average sensitivity value of obtained for the same feature using the k-NN classifier (89.2%). Feature 2 does not perform well for seizure detection with all classifiers, except with NB and CT classifiers, as can be seen from Figure 3 and Supplementary Table S2. In the same context, the averaged performance measures of accuracy, sensitivity and specificity obtained using all three features and five classifiers for both EMD and DWT based dictionaries are shown with upper and lower confidence bounds for the 95% confidence intervals in Figure 4. Significant variation of seizure detection results using Feature 2 with SVM classifier can also be observed from Figure 4.

If specificity is also taken into account, the best seizure detection performance is still obtained using Feature 3 with SVM classifier. This is not surprising, as SVM has been the classifier of choice for seizure detection [e.g., (24, 33, 47, 48)]. The seizure detection results obtained using Feature 3 and SVM classifier for the EMD-based dictionary approach in terms of the averaged values of accuracy, sensitivity and specificity are 86.5, 89.9, and 86.4%, respectively. Analyzing the patient-specific seizure detection results (Supplementary Table S3), it can be seen that the sensitivity value of patient 4 is very low at 34.8%, although with a high specificity of 91.1%. For patient 2, which has a sensitivity value of 100%, the specificity value is low at 46.9% (part of EEG recordings of patient 2 from the channel selected for seizure detection are shown in Figure 1). Patient number 12 is another patient with a relatively low sensitivity value at 81.3%, and a lower specificity value of 60.5%, whereas patient 13, while having a higher sensitivity value of 87.3%, also has a lower specificity value of 78.2%. Patient 17 is another patient with a specificity value lower than 80%, with a value of 78.2%, though with a high value of sensitivity at 94.4%.

While the sensitivity for patient 4 (part of EEG recordings from the channel selected for seizure detection are shown in Figure 1) is quite low with a high specificity with Feature 3 and SVM classifier for EMD-based dictionary approach, the values for the same feature and classifier using the DWT-based dictionary approach are 52.2 and 86.9% for sensitivity and specificity, respectively. This shows that for this patient, the DWT-based dictionary is better able to capture the structure of the seizures. However, there appears to be a trade-off between sensitivity and specificity for this patient. If Feature 1 is considered for this patient, the seizure detection performance is considerably improved with a sensitivity value of 93.5% and specificity value of 60.8% with SVM classifier using DWT-based dictionary. On the other hand, using the linear LDA classifier considerable improvement is obtained in the specificity (73.3%) with only slight loss in sensitivity (91.3%), again with DWT-based dictionary. Similar values of sensitivity and specificity are obtained for the same feature using NB classifier, and the difference between EMD and DWT-based dictionaries is also small.

The trade-off between sensitivity and specificity values can also be seen for other patients as well, such as patient 2, which has a high sensitivity value (100%) but a low specificity value (46.9%) obtained using Feature 3 with SVM classifier as shown in Table 2. However, for the same feature and EMD-based dictionary, there is considerable improvement in the specificity value, but at the expense of sensitivity, for CT classifier (85.7% sens. and 92.7% spec.). Although some patients show better seizure detection performance with some feature or classifier, the seizure detection performance of other patients with that particular feature and classifier is low, thereby demonstrating the challenge presented by the data of these patients for seizure detection.

**Table 2:** Patient-specific classification results in terms of accuracy, sensitivity and specificity, obtained using EMD-based dictionary approach with Feature 3 (reconstruction error ϵ) and SVM classifier.

**Patient No**.	**Channel No**.	**AUC**	**Accuracy (%)**	**Sensitivity (%)**	**Specificity (%)**
1	14	0.98	94.6 (99.6, 98.6)	90.9 (99.4, 97.3)	94.7 (99.6, 98.7)
2	21,11,2	0.88	89.2 (94.0, 94.4)	90.5 (99.2, 100)	89.2 (93.9, 94.3)
3	20	0.97	87.9 (93.4, 92.0)	100.0 (100, 100)	87.8 (93.3, 91.9)
4	21,12,11	0.71	83.3 (97.7, 83.2)	50.0 (100, 96.5)	83.5 (97.6, 83.1)
5	12	0.99	91.9 (98.5, 98.9)	97.1 (91.5, 82.9)	91.8 (98.7, 99.5)
6	20	0.96	86.3 (99.8, 67.7)	94.1 (0, 81.1)	86.3 (100, 67.7)
7	15	0.99	98.3 (99.3, 99.2)	87.5 (93.9, 85.8)	98.4 (99.4, 99.3)
8	11	0.95	80.7 (96.2, 96.1)	99.1 (77.2, 82.8)	80.1 (97.2, 96.8)
9	9	0.99	98.2 (98.1, 96.8)	88.2 (97.21, 98.1)	98.2 (98.2, 96.8)
10	4	0.94	88.4 (96.7, 98.9)	94.6 (95.6, 86.2)	88.4 (96.7, 98.9)
11	6	0.99	98.8 (99.7, 94.8)	93.0 (96.5, 98.5)	99.0 (99.9, 94.5)
12	16,13,12	0.75	66.2 (95.5, 80.5)	74.8 (92.7, 73.5)	66.1 (95.6, 80.7)
13	6,1,9	0.86	76.0 (91.7, 94.1)	94.6 (97.9, 96.4)	75.8 (91.6, 94.0)
14	14	0.92	85.9 (90.1, 92.0)	90.0 (98.5, 97.7)	85.9 (90.1, 91.9)
15	8	0.90	82.0 (NR, NR)	93.2 (NR, NR)	81.8 (NR, NR)
16	15	0.97	93.8 (79.9, 88.2)	87.5 (91.2, 73.7)	93.6 (79.7, 88.2)
17	18,13,10	0.93	79.9 (95.7, 96.2)	97.2 (99.6, 86.0)	79.7 (95.6, 96.5)
18	21	0.95	83.8 (75.8, 80.2)	97.4 (100, 100)	83.7 (75.4, 79.9)
19	6	0.97	95.6 (97.8, 98.4)	86.2 (96.1, 75.4)	95.7 (97.8, 98.9)
20	21	0.99	97.8 (94.3, 98.1)	94.4 (99.6, 94.2)	97.9 (94.2, 98.1)
21	10	0.87	81.0 (98.3, 98.5)	83.3 (98.7, 99.3)	81.0 (98.3, 98.5)
22	9	0.99	97.7 (97.5, 98.6)	96.0 (89.7, 97.4)	97.7 (97.7, 98.6)
23	8	0.98	90.3 (99.5, 98.8)	98.1 (96.3, 59.8)	90.3 (98.6, 99.4)
24	NR	NR	NR (99.6, 97.1)	NR (85.9, 67.3)	NR (99.7, 97.5)
Average		0.93	88.2 (95.1, 93.1)	90.3 (91.1, 88.3)	88.1 (95.2, 93.2)

*The values in brackets correspond to Zabihi et al. (11, 13), respectively, which are two recent works using a classifier training approach similar to ours with 25% training data (25% of seizure and non-seizure data, balanced using random under-sampling in Zabihi et al. (13), whereas our approach uses 30% of seizure data, and same amount of non-seizure data). NR means not recorded*.

**Figure 5:**
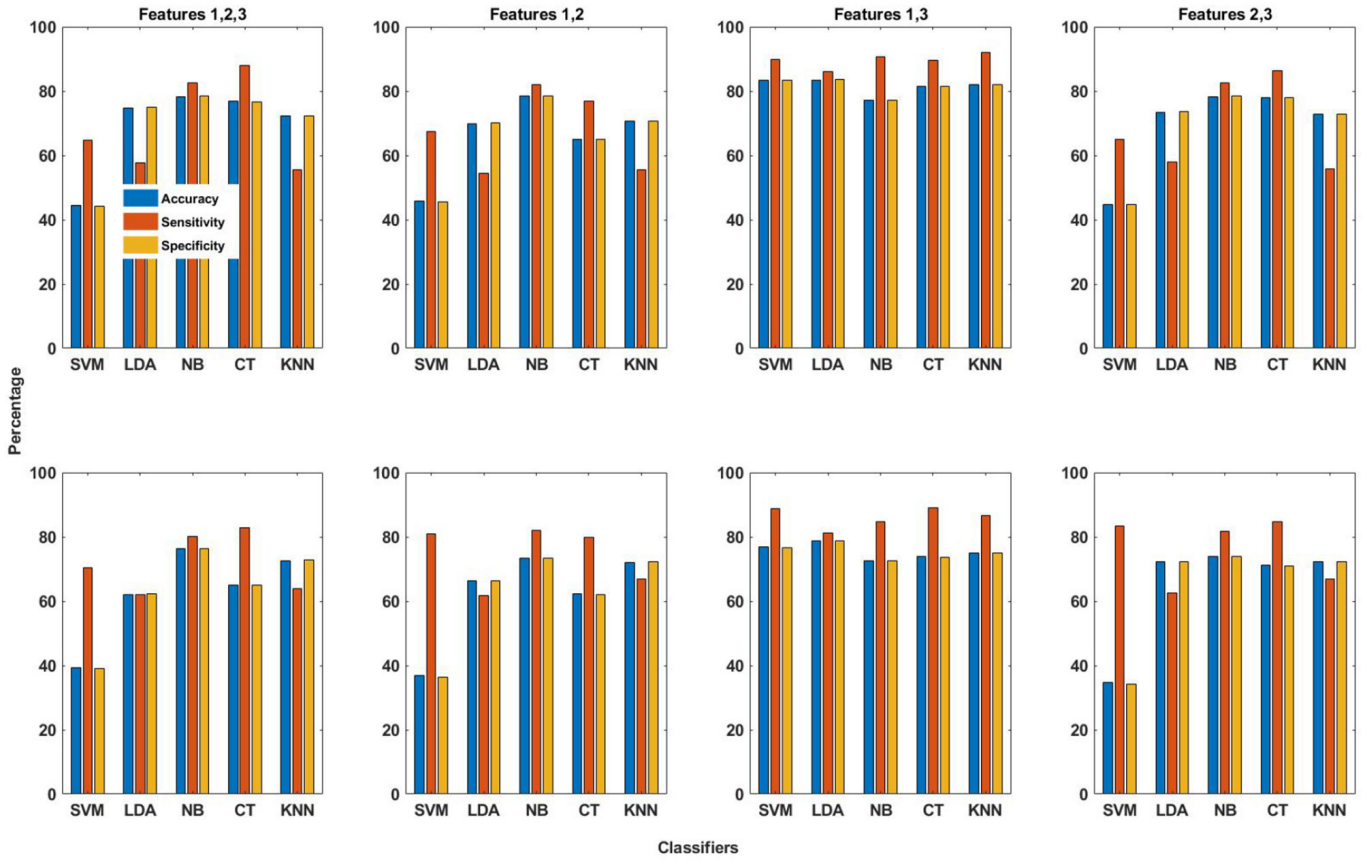
The averaged performance measures of accuracy, sensitivity and specificity obtained using combinations of all three features and five classifiers for EMD (top row in figure) and DWT (bottom row in figure) based dictionaries.

The averaged seizure detection results obtained using different combinations of the three features with EMD and DWT-based dictionaries both are shown in Figure 5. The values of accuracy, sensitivity and specificity obtained using EMD-based dictionary are in general higher than those obtained using DWT-based dictionaries. The best seizure detection results are obtained using the combination (F1,F3), whereby Feature 1 and Feature 3 are combined (section 2.5.4). Using this combination and EMD-based dictionary, the averaged values of accuracy, sensitivity and specificity obtained using SVM classifier are 83.3, 89.8, and 83.2%, respectively, whereas the k-NN classifier gives the highest averaged value of sensitivity, which is 91.9%, with values of 82.0 and 81.9% for accuracy and specificity, respectively. Although high averaged values of sensitivity are obtained using the combination (F1 and F3) and SVM and k-NN classifiers, the values of specificity are relatively lower, compared to the values obtained using Feature 3 with SVM classifier and EMD-based dictionary. When all features are combined (F1, F2, and F3), the seizure detection performance is in general not good, except for NB and CT classifiers, with similar pattern holding for combination of Feature 2 and 3 (F2 and F3). For Features 1 and 2 combined (F1 and F2), averaged seizure detection results are quite low, except for NB classifier.

As already mentioned, the best seizure detection performance is obtained using the SVM classifier with Feature 3 and EMD-based dictionary, with averaged values of accuracy, sensitivity and specificity given by 86.5, 89.9, and 86.4%, respectively. We have already mentioned in this section that patients 2, 4, 12, 13, and 17 have comparatively low sensitivity or specificity values (less than 80%) for this combination of dictionary, feature and classifier. For these patients, we apply the extension of the channel selection technique described in section 2.6 to improve the seizure detection performance. For patient 2, application of this extended channel selection technique results in improvement of specificity from 46.9 to 89.2%, though at the cost of decrease in sensitivity from 100 to 90.5%. For patient 4, the sensitivity value increases from 34.8 to 50%, but is accompanied by a decrease in specificity from 91.1 to 83.5%. For patient 13, the specificity value decreases slightly from 78.2 to 75.8%, but the sensitivity value increases to 94.6% from 87.3%. In the case of patient 17, there is an increase in sensitivity and specificity both, from 94.4 to 97.2%, and 79.7% from 78.6%, respectively. For patient 12, however, increase in specificity to 66.1% from 60.5% is accompanied by a decrease in sensitivity to 74.8% from 81.3%, thereby representing the case where improvement in one performance measure is obtained at the cost of similar decrease in another performance measure.

The seizure detection results obtained using Feature 3 and SVM classifier for the EMD-based dictionary approach and this extended channel selection technique for patients 2, 3, 12, 13, and 17 are shown in Table 2 for all 23 patients, which also lists the channel(s) used for each patient and the associated AUC. The averaged values of accuracy, sensitivity and specificity are improved to 88.2, 90.3, and 88.1%.

In order to rule out any bias in the seizure detection results, we validated the results reported in Table 2 using 5-fold cross-validation with the same testing data for each patient, and obtained values of 90.3, 93.5, and 87.12% for accuracy, sensitivity and specificity, respectively. The seizure detection results were obtained as before, by selecting the channel with the best value of AUC. For some patients, the channels selected in this case were different from the ones reported in Table 2, however these belong to the same region of the brain. This demonstrates that the seizure detection approach demonstrated here is robust, as for k-fold cross-validation, the data is divided into k-folds randomly, which are then used for training and testing.

As mentioned earlier in section 2.4, our approach does not require any pre-processing. In order to verify this experimentally, we bandpass filtered the EEG records between 1 and 60 Hz before segmentation (using a second order Butterworth bandpass filter), as in Zabihi et al. (13). The difference in seizure detection results was found to be insignificant.

### 3.3. Computational Time Requirements

All the experiments reported in this paper were implemented using MATLAB version 2019b on a desktop computer with a 2.40 GHz processor and 16GB of RAM. The computational time requirements were measured in terms of time required for EMD and DWT decomposition, EMD and DWT-based dictionary training, feature extraction, classifier training and classification.

The runtime per segment (4 s length) is measured, which is then averaged over all channels of all patients. The decomposition time for one segment using DWT is 7.8 ms compared to 71.8 ms using EMD. This is not surprising, given that EMD is an iterative algorithm, implementation of which is time intensive. However, since dictionaries can be created independent of seizure detection, the comparatively higher computational time for EMD-based dictionary creation does not preclude its use in a practical scenario.

The time for dictionary training for one segment is 0.88 ms on average for DWT-based approach compared to 1.10 ms for EMD-based approach. This difference can be explained in terms of the lower number of iterations required for training DWT-based dictionaries, as discussed in section 3.1.

The time required for feature extraction in terms of Feature 2 (coefficient vector) and Feature 3 (reconstruction error) is 0.34 ms and 0.41 ms on average for one segment, respectively, for DWT-based dictionary. The corresponding times for EMD-based dictionary are 0.39 and 0.46 ms per segment. Extraction of Feature 1 (projections coefficients) feature is more computationally expensive, with 7.9 ms per 4-s segment for DWT-based dictionary compared to 8.6 ms for EMD-based dictionary, due to the iterative nature of projecting testing segments against the trained dictionary, as in the current implementation the testing segments are projected one by one against the trained dictionary. The slight difference in computational time for the EMD and DWT-based dictionaries can be attributed to the smaller size of DWT-based dictionaries on average, as reported in Table 1.

For classifier training and testing, we report the computational time required by Feature 3 (reconstruction error) and SVM classifier, as we have reported seizure detection results based on this combination in Table 2. The time required for SVM classifier training is 89 ms, whereas the time required for classification is 0.47 ms, for 1-h of EEG recording. The time required for classifier training and testing for the other classifiers is much less than that required by the non-linear SVM. The computational times reported here are also summarized in Table 3.

**Table 3:** Computational time requirements of the proposed methodology for 1 h EEG recording.

**Dictionary training**		**Feature extraction**						**Classifier training and testing**	
**EMD**	**DWT**	**Feature 1**		**Feature 2**		**Feature 3**		**EMD Dictionary, Feature 3, SVM Classifier**	
		**EMD**	**DWT**	**EMD**	**DWT**	**EMD**	**DWT**	**Classifier training**	**Seizure detection**
0.99 s	0.79 s	8.6 s	7.1 s	0.35 s	0.31 s	0.41 s	0.37 s	89 ms	0.47 ms

## 4. Discussion

### 4.1. Effect of Mother Wavelet

Many previous seizure detection studies have used the Debauchies family of mother wavelets when decomposing EEG signals, with the db4 mother wavelet most commonly used (2, 36, 46, 48, 49). Some studies have used other mother wavelets as well, such as db5 (3), db6 (5, 50), sym4 (51) or the Mexican Hat (47). However, the reason for using any of the particular mother wavelets is never mentioned, except for the study in Tian et al. (36), which considers higher order Debauchies mother wavelets to be better suited for EEG signals, but more computationally expensive, and hence settles for db4. A study presented in Rafiee et al. (52) tests a number of mother wavelet functions across different signals, and considers db44 to be the most similar mother wavelet functions across a variety of biological signals. In this context, the study in Al-Qazzaz et al. (53) considers the sym9 to be most similar to EEG signals recorded during a working memory task, but also finds db7 to be very similar to the recorded EEG signals.

As already mentioned in section 2.3, for this study we used the db4 mother wavelet. However, we also tested our approach with db6 mother wavelet in order to quantify the difference between db4 and db6 mother wavelets in the context of seizure detection using the proposed approach. In this context, it was observed that the seizure detection performance using these mother wavelets is quite similar, and an increase in sensitivity/specificity is accompanied by a decrease in specificity/sensitivity, particularly for Feature 1 and Feature 2. For Feature 3, however, there is increase in both sensitivity and specificity using db4 with all classifiers except CT classifier. These performance aspects can also be seen from Supplementary Figure S1. One other difference between using db4 and db6 mother wavelets relates to lesser dispersion of the performance measure values around the mean when using db4 compared to db6 mother wavelet.

### 4.2. Use of Different Features

The seizure detection performance of all features obtained using both EMD and DWT-based dictionaries with all classifiers in terms of averaged performance measures of accuracy, sensitivity and specificity are shown in Figure 3, and Supplementary Tables S1–S3. It may be observed from this figure and tables that for Feature 1, there is small difference in values of the performance measures for EMD and DWT-based dictionaries. For Feature 2, the averaged sensitivity values are in general greater for DWT-based dictionaries. On the contrary, for Feature 3, the averaged sensitivity values are higher in general for EMD-based dictionaries, with a value greater than 80% for all classifiers. Feature 3 therefore turns out to be the best performing of all features with all classifiers.

We have shown that all three features, which have been used in traditional approaches for dictionary learning, can be used for seizure detection with the data-driven dictionary approach using both EMD and DWT-based dictionaries. From among the features, the best seizure detection performance has been obtained using Feature 3, namely the reconstruction error, and EMD-based dictionary. An EMD-based dictionary, consisting of atoms composed of IMFs that have been decomposed using a data-driven algorithm, instead of using a basis function, is better able to capture the distinctive characteristics of seizures in EEG signals. However, a DWT-based dictionary also performs well, and can be of practical use in a seizure detection scenario as well.

### 4.3. Comparison With Other Approaches

Due to high inter and intra-patient seizure variation in the long-term recordings of the CHB-MIT EEG database, as well as contamination by sleep and physiological artifacts, not many studies have used this database due to the adverse affect of these factors on seizure detection performance (11).

In Table 2, we have directly compared seizure detection results of this study with the results of a recent study, where the seizure detection methodology has been structured in the same way using early part of the data as training data, and the later part as testing data (13), as well as a previous study by the same authors (11). Both of these studies use 25% of the data for training. However, the study in Zabihi et al. (13) uses 25% of the early seizure as well as non-seizure data, and then balances the data using random under-sampling, as the number of seizure segments is much less than the non-seizure segments. On the other hand, our approach uses 30% of the seizure segments, and then selects an equal number of non-seizure segments. In the CHB-MIT database, the lengths of recordings with seizures vary for each patient, and for all patients, seizure recordings are much smaller than non-seizure recordings. As a result, the actual data used for training will be much less in our approach.

The seizure detection results obtained using our approach compare very well with the results of the two approaches reported in the Table 2, despite using the smallest amount of data for classifier training.

The difference in averaged sensitivity value obtained using our approach and that in Zabihi et al. (13) is small, at 90.3% against 91.1%. The sensitivity value is an adequate comparison metric, as detection of all seizures at the cost of more false alarms is preferable to missing seizures with less false alarms (54). Furthermore, it has also been pointed out in Zabihi et al. (13) that accuracy is not a reliable performance metric due to unbalanced numbers of seizure and non-seizure segments. Furthermore, the work in Zabihi et al. (13) uses a two-layer classification scheme, using LDA classifiers per channel in the first layer, followed by an artificial neural network, and incorporates a post-processing consisting of morphological filtering as well. The methodology of our classification scheme, in contrast, is much simpler, and the computational requirements, as described in section 3.3, are meager.

In Table 4, the seizure detection results obtained using EMD-based dictionary approach with Feature 3 and SVM classifier are compared with some recently reported studies on patient-specific seizure detection. This comparison is in terms of the performance measures of accuracy, sensitivity and specificity. The seizure detection results of this study compare very well with studies using similar training/testing partitions. Other studies, such as Bhattacharyya and Pachori (7), Kaleem et al. (8), and et al. (28) have used hand-engineered features or k-fold cross-validation techniques with data balancing, which might not hold well in a practical scenario. Furthermore, the approach in this paper does not require feature processing before classification and post-processing of the results after classification to obtain seizure detection, such as in Bhattacharyya and Pachori (7). Additionally, as mentioned before, the proposed approach uses the smallest amount of data for classifier training of all the other approaches. Another aspect that distinguishes our approach is that although the training and testing data have been selected by maintaining the order in time, with training data having occurred earlier than testing data, our approach is robust to changes in this order, as demonstrated using 5-fold cross validation mentioned in section 3.2.

**Table 4:** Comparison with some other patient-specific seizure detection methods using the CHB-MIT EEG database.

			**Results**		
**Reference**	**Patients**	**Training set (%)**	**Av. Sen (%)**	**Av. Spec. (%)**	**Av. Acc. (%)**
Bhattacharyya and Pachori (7)	All (except 12)	10-fold CV	97.9	99.6	99.4
Kaleem et al. (8)	All	5-fold CV	99.8	99.6	99.6
Kaleem et al. (28)	All	5-fold CV	94.3	91.5	92.9
Zabihi et al. (11)	All (except 15)	50% of seizure and non-seizure	89.1	94.8	94.7
Zabihi et al. (11)	All (except 15)	25% of seizure and non-seizure	88.3	93.2	93.1
Zabihi et al. (13)	All (except 15)	25% of seizure and non-seizure, balanced using random undersampling technique	91.1	95.2	95.1
This work	All	30% of seizure, and equal amount of non-seizure	90.3	88.1	88.2

We would also like to point out that some other recent studies not listed in Table 4, such as Li et al. (55) and Mehla et al. (33), also report very high values of the performance measures of accuracy, sensitivity and specificity for the same EEG database. However, the seizure detection methodology in such studies is also constructed in a way which may not be conducive in a practical scenario, as mentioned in the previous paragraph. For example, the study in Li et al. (55) balances the data using an over-sampling technique by adding synthetic data, whereas the study in Mehla et al. (33) selects 1 h long records randomly from seizure and non-seizure data of all patients combined. In this regard, we would like to reiterate our position that our proposed approach caters to the realistic scenario of using a small portion of patients' historical data for the steps that lead to seizure detection using newer data. Fair comparison of our approach, therefore, is to the very few studies that are constructed as such, such as the ones listed in Table 2. As we show next, our proposed approach outperforms the approaches listed in Table 2 in terms of computational efficiency.

Compared to the computational time requirements of the methodology presented in Zabihi et al. (13), which requires around 38 s for pre-processing, feature extraction, classifier training and classification for 1-h EEG recording, our presented approach requires around 1.5 s for dictionary creation, feature extraction, classifier training and classification. There is also a 9 s gap required for post-processing after each segment in the approach of Zabihi et al. (13), which would significantly add to the overall computational time requirements. The proposed approach is therefore significantly efficient and thus practical than the approach presented in Zabihi et al. (13).

For seizure detection, we use all the available seizure and non-seizure testing data. For each patient, the number of non-seizure testing segments is significantly more than seizure testing segments. The seizure detection results we have presented in Table 2 have been obtained using the unequal testing data. If, however, we use the same number of seizure and non-seizure testing segments with the EMD-based dictionary approach with Feature 3 and SVM classifier, we obtain the values of accuracy, sensitivity and specificity given by 93.0, 90.9, and 95.1%, respectively. These values of the performance measures are obtained when the same number of non-seizure segments as the seizure segments are taken from the available testing segments while maintaining the order in time. However, even if the same number of non-seizure testing segments are selected randomly, the change in values of the performance measures is negligible. Therefore, a practical seizure detection scenario could involve using equal-sized seizure and non-seizure data. Furthermore, as more and more data for each patient becomes available, the size of the training data could be increased, and we expect the proposed method to provide even better seizure detection performance, also for unequal seizure and non-seizure testing data.

### 4.4. Limitations of the Study

Since the proposed method requires seizure and non-seizure data for dictionary creation and learning, and classifier training, patients with few and short duration seizures would represent a challenge to the proposed methodology, specially for classifier training, which happens for each EEG channel individually, contrary to the dictionary creation and learning stage, where data of all channels is combined. This situation could be dealt with by decreasing the segment length, which is currently 4 s. However, this may lead to a decrease in the seizure detection performance, as discussed in section 2.1. Another option could be to combine the data of all EEG channels, as opposed to having seizure detection per channel.

#### 4.4.1. Limitations of EMD

While DWT has a rich mathematical formulation, and the decomposition behavior can be studied precisely, EMD is an empirical technique that does not have a mathematical formulation. Although numerous extensions of the EMD algorithm have been introduced that formulate the basic EMD algorithm in a formal framework [e.g., the method described in Dragomiretskiy and Zosso (56), or the many methods referenced in Stallone et al. (43)], such extensions require numerous parameters to be set, often heuristically (57), which in our view defeats the purpose of a model-free and data-driven decomposition. Therefore, we have used the EMD algorithm in its basic form.

However, there are certain limitations of EMD which have to be kept in view. One of these is the effect of boundary conditions, or end effects on the decomposition process, whereby, due to the finite length of the signal, the sifting process in the EMD algorithm may result in anomalously high amplitudes and spurious wave peaks for the IMFs (specially higher order IMFs) near the boundaries (43). Numerous methods are available to mitigate the end effects; the implementation of EMD algorithm that we use mirrors the signal extrema near the boundaries with good results (40). Furthermore, due to the nature of the dictionary learning algorithm (section 2.3), only those atoms (IMFs) are added to the trained EMD-based dictionary which are most similar to the dictionary training signals, thereby significantly reducing the chance of any atoms with spurious decomposition artifacts being added to the trained dictionary.

The other important aspect to be considered is the effect of mode-mixing. IMFs are considered to be zero-mean amplitude and frequency modulated components. Mode-mixing refers to the phenomenon whereby different frequency components in the original signal are decomposed into the same IMF, or the same frequency component is decomposed into different IMFs. Numerous methods for mitigating the effect of mode-mixing have been proposed (58). Due to mode-mixing, IMFs can become devoid of any physical meaning. However, as we have explained in section 2.4, our proposed approach does not rely on assigning a physical meaning to the decomposed components. Also, for the case of EMD-based dictionary, the task of seizure detection is not affected by mode-mixing, as both seizure and non-seizure signals will be affected by mode-mixing, if at all. Also, the DWT components are different from the definition of IMFs, and may also be similar to mode-mixed IMFs. Therefore, we do not consider mode-mixing to be detrimental to EMD-based dictionaries.

## 5. Conclusion

This paper has presented the signal-derived dictionary approach using both EMD and DWT-based dictionaries. Similar to the EMD-based approach, the DWT-based dictionary approach is also a viable approach, and benefits from a well-established and computationally fast decomposition approach. The use of three features used in traditional dictionary learning approaches obtained from EMD and DWT-based dictionaries has also been demonstrated with good seizure detection results, and compared for both types of dictionaries. These features, when used with different classifiers, are robust to variations of seizure types amongst patients, as seizures can be consistently detected across all patients with high sensitivity and specificity. This work has also shown that the signal-derived dictionary approach is robust to the use of a model-free, data-driven dictionary, as well as a model-based dictionary that requires an *a priori* basis. The current methodology is a patient-specific seizure detection methodology, though it could be extended to patient-independent seizure detection as well. This will require a complete re-design of the methodology, following which the seizure detection results could be cross-validated using a leave-one-out scheme, as implemented in Nagaraj et al. (22).

Despite using small amounts of data for classifier training, seizures were detected with a sensitivity of 90.3% and specificity of 88.1% over the EEG records of all 23 patients. The sensitivity rate of all patients is above 85%, except for 2 patients. This work represents one of the few works which caters to the realistic scenario where a small portion of the historical patient data is used for the methodology, including classifier training, and seizure detection is then performed using the newer data.

The proposed approach and the features used are also different from seizure detection using hand-engineered features. No pre-processing of data or feature processing of any kind is required for the proposed approach. Furthermore, all channels of EEG recordings are used for seizure detection, and the channel(s) with the best performance is selected based on the criterion of highest area under the receiver operating curve.

The computational requirements of different aspects of the overall methodology, including dictionary creation and training, feature extraction, classifier training and classification, are also practical compared to similar approaches. On the whole, using small amounts of patients' historical data for classifier training, the computational efficiency, and seizure detection with high sensitivity distinguish our approach from other available approaches for automatic seizure detection. Furthermore, as the proposed approach is a data-driven approach, as more and more data will be available, the performance of the proposed approach will continue to further improve.

An interesting direction of further work will involve a new model for signal-derived empirical dictionary approach, whereby class-specific dictionaries are learned, instead of learning a combined dictionary. The features extracted from class-specific trained dictionaries can be expected to contain characteristics representing similarities with one class as well as dissimilarities with the other class, and hence contain more discriminatory information. Furthermore, the methodology presented in this paper can be categorized as a machine learning approach. An interesting future direction will consist of incorporating deep learning for automatic seizure detection using the signal-derived empirical dictionary approach.

## Data Availability Statement

Publicly available datasets were analyzed in this study. This data can be found here: https://physionet.org/content/chbmit/1.0.0/.

## Author Contributions

MK: conceptualization, investigation, methodology, software, and writing—original draft. AG and SK: supervision. SK: writing—review and editing. All authors contributed to the article and approved the submitted version.

## Funding

This work was supported by the Natural Sciences and Engineering Research Council of Canada (NSERC).

## Conflict of Interest

The authors declare that the research was conducted in the absence of any commercial or financial relationships that could be construed as a potential conflict of interest.

## Publisher's Note

All claims expressed in this article are solely those of the authors and do not necessarily represent those of their affiliated organizations, or those of the publisher, the editors and the reviewers. Any product that may be evaluated in this article, or claim that may be made by its manufacturer, is not guaranteed or endorsed by the publisher.
