# Supplementary material for: Comparison of Empirical Mode Decomposition, Wavelets, and Different Machine Learning Approaches for Patient-Specific Seizure Detection Using Signal-Derived Empirical Dictionary Approach
Source: Front Digit Health. 2021 Dec 13;3:738996. doi: 10.3389/fdgth.2021.738996 (PMC8710482; doi:10.3389/fdgth.2021.738996)
Supplement: Supplementary file 1 [file Data_Sheet_1.PDF]

Supplementary Material

1 SUPPLEMENTARY TABLES AND FIGURES

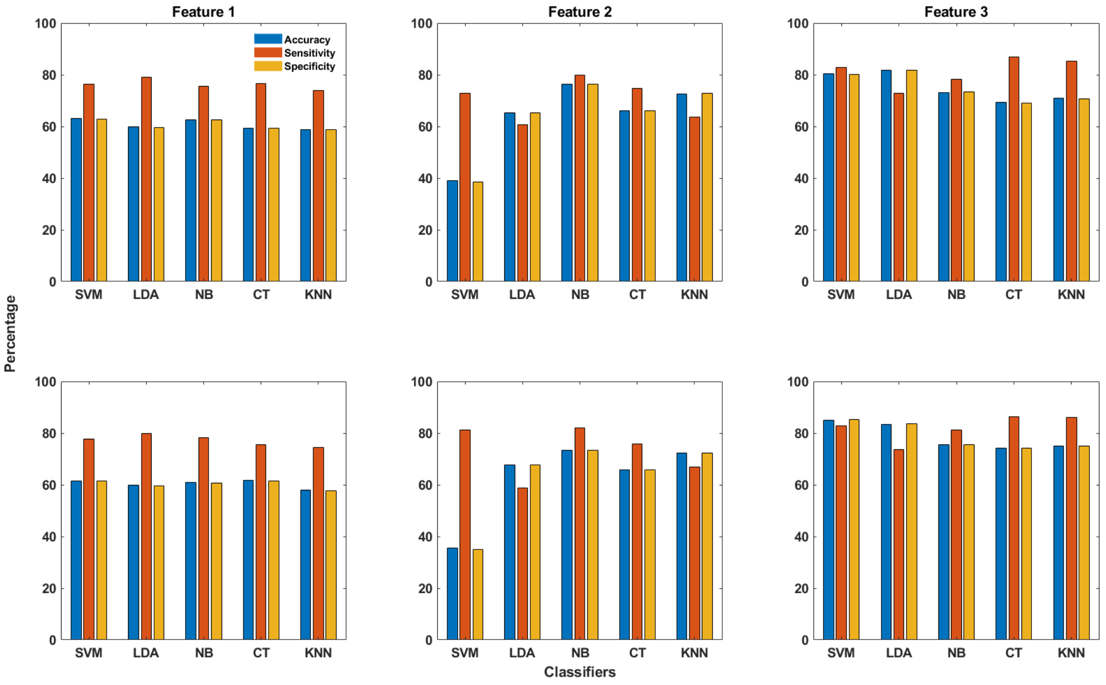

Figure S1. The averaged performance measures of accuracy, sensitivity and specificity obtained using all three features and five classifiers for DWT based dictionary using db6 mother wavelet (top row in figure) and db4 mother wavelet (bottom row in figure).

Table S1. Patient-specific classification results using Feature 1 in terms of performance measures of accuracy (Acc.), sensitivity (Sens.) and specificity (Spec.), obtained using EMD and DWT-based dictionaries and 5 classifiers.

Pat.	SVM						LDA						NB						CT						k-NN					
No	Acc.		Sens.		Spec.		Acc.		Sens.		Spec.		Acc.		Sens.		Spec.		Acc.		Sens.		Spec.		Acc.		Sens.		Spec.	
	EMD	DWT	EMD	DWT	EMD	DWT	EMD	DWT	EMD	DWT	EMD	DWT	EMD	DWT	EMD	DWT	EMD	DWT	EMD	DWT	EMD	DWT	EMD	DWT	EMD	DWT	EMD	DWT	EMD	DWT
1	45.7	59.1	80.0	69.1	45.4	59.0	41.8	47.6	85.4	81.8	41.4	47.2	42.3	48.6	85.4	80.0	41.9	48.3	40.5	40.1	83.6	83.6	40.1	39.7	42.3	43.9	76.4	78.2	41.9	43.6
2	72.6	48.6	80.9	85.7	72.5	48.2	39.3	38.9	95.2	90.5	38.7	38.4	39.5	39.1	95.2	90.5	38.9	38.6	61.0	62.8	85.7	85.7	60.7	62.6	69.9	42.8	57.1	100	70.0	42.2
3	57.1	42.8	78.0	86.0	57.0	42.4	68.1	63.4	74.0	68.0	68.0	63.4	57.5	57.8	78.0	78.0	57.3	57.6	72.8	44.2	54.0	82.0	72.9	43.9	54.1	49.1	78.0	74.0	53.9	48.8
4	59.2	61.0	93.5	93.5	59.1	60.8	72.3	73.4	89.1	91.3	72.2	73.3	75.9	73.4	89.1	86.9	75.9	73.3	71.9	68.9	69.6	78.2	71.9	68.8	69.2	59.6	71.7	82.6	69.2	59.5
5	93.3	88.5	61.4	64.3	93.8	88.9	91.3	81.9	65.7	74.3	91.7	82.1	92.3	82.2	64.3	71.4	92.8	82.4	88.5	85.3	60.0	61.4	88.9	85.7	77.8	73.4	67.1	68.6	77.9	73.5
6	70.0	58.8	82.3	82.3	70.0	58.7	58.8	52.2	94.1	88.2	58.8	52.1	68.9	56.4	76.5	82.3	68.9	56.4	59.6	70.5	94.1	76.4	59.6	70.5	56.1	48.9	70.6	82.3	56.1	48.8
7	90.9	90.9	45.0	45.0	91.2	91.1	60.5	63.5	80.0	77.5	60.4	63.4	84.8	82.2	60.0	57.5	84.9	82.3	70.2	66.3	72.5	67.5	70.2	66.3	63.7	57.5	72.5	72.5	63.6	57.4
8	72.9	71.6	88.6	89.5	72.5	71.1	80.1	74.2	67.5	85.9	80.5	73.9	77.7	73.8	74.6	87.7	77.8	73.5	74.8	67.8	81.6	89.4	74.7	67.2	73.9	70.8	80.7	86.8	73.8	70.4
9	3.8	3.66	97.1	100	3.4	3.2	4.8	9.3	100	100	4.5	8.9	25.9	6.3	94.1	100	25.6	5.9	25.6	23.2	91.2	85.3	30.5	22.9	28.7	22.0	91.2	85.3	28.5	21.8
10	87.0	90.6	80.0	67.3	87.1	90.7	85.1	85.4	80.0	70.9	85.1	85.5	85.0	84.8	80.0	70.9	85.0	84.8	82.9	76.9	74.5	65.4	83.0	76.9	78.5	74.4	76.4	67.3	78.5	74.4
11	70.6	59.8	69.0	78.0	70.7	58.9	49.9	52.8	90.0	89.0	48.1	51.2	62.8	74.5	81.0	68.0	62.0	74.8	74.6	76.6	53.0	53.0	75.6	77.7	73.8	74.3	52.0	52.0	74.8	75.4
12	55.5	55.0	78.1	82.9	55.1	54.6	73.6	74.9	66.7	58.5	73.7	75.2	49.2	60.8	81.3	78.0	48.8	60.5	54.2	58.6	66.7	67.5	53.9	58.5	66.1	57.8	60.2	73.2	66.2	57.6
13	70.9	83.4	83.6	69.1	70.8	83.5	67.7	75.1	90.9	80.0	67.5	75.0	74.3	81.7	83.6	69.1	74.2	81.8	53.6	77.6	87.3	67.3	53.3	77.6	51.9	65.3	87.3	72.7	51.6	65.2
14	82.2	87.2	60.0	50.0	82.3	87.3	63.5	62.5	60.0	65.0	63.6	62.5	80.8	75.8	60.0	55.0	80.8	75.9	81.8	82.0	70.0	85.0	81.8	82.0	59.3	54.5	85.0	75.0	59.3	54.5
15	70.6	69.0	60.1	62.1	70.8	69.1	44.3	46.4	74.2	73.4	43.7	45.9	42.5	45.9	75.8	73.4	41.8	45.4	53.7	55.1	70.6	64.1	53.4	54.9	54.1	48.7	69.8	65.3	53.7	48.4
16	70.1	80.5	37.5	50.0	70.1	80.6	7.6	8.2	100	100	7.5	8.0	11.5	6.8	100	100	11.4	6.6	28.4	62.6	100	75.0	28.3	62.6	25.2	73.5	100	62.5	25.1	73.5
17	54.5	27.9	86.1	97.2	54.0	27.0	70.0	69.1	66.7	63.9	70.1	69.2	64.5	62.0	72.2	66.7	64.4	61.9	66.4	51.0	52.8	72.2	66.6	50.7	61.4	52.2	66.7	72.2	61.3	51.9
18	37.9	38.6	84.6	92.3	37.6	38.2	63.4	65.0	61.5	61.5	63.4	65.1	42.6	42.2	76.9	82.0	42.3	41.9	61.7	41.6	66.7	89.7	61.7	41.2	58.9	38.5	64.1	87.2	58.9	38.1
19	91.8	92.0	86.2	65.5	91.8	92.3	89.7	90.6	86.2	65.5	89.7	90.9	89.7	90.6	86.2	65.5	89.7	90.9	81.1	55.2	86.2	93.1	81.1	54.8	82.8	84.1	82.8	75.9	82.8	84.2
20	77.4	61.4	69.4	77.8	77.5	61.3	68.7	60.7	66.7	72.2	68.7	60.6	84.7	76.4	66.7	72.2	84.8	76.4	51.8	77.6	80.6	58.3	51.6	77.7	56.8	60.7	66.7	75.0	56.7	60.6
21	46.8	36.6	100	100	46.5	36.1	51.3	51.8	87.5	100	51.1	51.5	50.2	48.7	87.5	100	49.9	48.3	44.4	43.2	100	91.7	44.0	42.9	45.7	46.0	79.2	79.2	45.5	45.7
22	61.6	50.1	88.0	96.0	61.4	49.7	68.4	63.3	80.0	96.0	68.3	63.0	70.5	60.9	80.0	96.0	70.4	60.5	62.3	73.6	84.0	76.0	62.1	73.6	63.9	71.1	84.0	68.0	63.7	71.1
23	74.5	58.6	53.8	82.7	74.6	58.4	64.1	64.7	71.1	80.8	64.0	64.6	60.8	69.6	71.1	71.1	60.8	69.6	66.2	57.3	53.8	67.3	66.3	57.3	52.3	62.3	69.2	57.7	52.2	62.3
Mean	65.9	61.5	75.8	77.7	65.9	61.4	60.2	59.8	79.7	79.7	60.0	59.6	62.3	60.9	79.1	78.4	62.2	60.8	62.3	61.7	75.6	75.4	62.3	61.6	59.4	57.9	74.3	74.5	59.4	57.8
std	20.2	22.7	16.3	16.4	20.3	23.0	21.9	20.5	12.5	13.0	22.2	20.7	21.5	22.5	10.6	13.0	21.7	22.7	16.1	15.8	14.8	11.4	16.3	16.0	14.7	14.5	11.3	10.4	14.8	14.7

Table S2. Patient-specific classification results using Feature 2 in terms of performance measures of accuracy (Acc.), sensitivity (Sens.) and specificity (Spec.), obtained using EMD and DWT-based dictionaries and 5 classifiers.

Pat.	SVM						LDA						NB						CT						k-NN					
No	Acc.		Sens.		Spec.		Acc.		Sens.		Spec.		Acc.		Sens.		Spec.		Acc.		Sens.		Spec.		Acc.		Sens.		Spec.	
	EMD	DWT	EMD	DWT	EMD	DWT	EMD	DWT	EMD	DWT	EMD	DWT	EMD	DWT	EMD	DWT	EMD	DWT	EMD	DWT	EMD	DWT	EMD	DWT	EMD	DWT	EMD	DWT	EMD	DWT
1	30.8	54.9	100	100	30.1	54.5	84.6	86.6	58.2	45.4	84.8	86.9	87.1	89.9	98.2	92.7	87.0	89.9	62.5	73.5	90.9	87.3	62.3	73.4	85.6	85.5	45.4	52.7	85.9	85.9
2	98.9	44.5	0	80.9	100	44.1	70.9	71.8	52.4	42.8	71.1	72.1	53.6	48.1	100	95.2	53.1	47.6	62.3	51.5	66.7	80.9	62.2	51.2	90.0	68.6	38.1	52.4	90.6	68.7
3	99.2	21.7	0	100	100	21.1	93.9	92.5	48.0	64.0	94.2	92.8	80.7	83.3	96.0	98.0	80.6	83.2	75.9	81.0	92.0	86.0	75.8	81.0	94.3	90.4	56.0	76.0	94.6	90.5
4	52.9	7.5	89.1	100	52.8	7.1	85.6	79.7	32.6	39.1	85.8	79.8	23.5	23.4	39.1	34.8	23.4	23.3	58.6	73.6	56.5	67.4	58.6	73.6	88.2	80.9	47.8	65.2	88.4	81.0
5	98.4	52.7	0	100	100	51.9	80.1	86.8	55.7	52.8	80.5	87.3	89.6	82.5	81.4	85.7	89.7	82.4	79.7	70.9	77.1	91.4	79.7	70.5	94.5	89.2	34.3	71.4	95.5	89.6
6	0.08	15.7	100	100	0.01	15.6	52.4	35.6	64.7	82.3	52.4	35.5	82.2	83.8	94.1	94.1	82.2	83.8	37.1	50.2	88.2	82.3	37.1	50.1	14.4	20.0	100	100	14.3	20.0
7	3.9	5.4	100	100	3.5	4.9	93.0	86.2	47.5	50.0	93.2	86.3	82.8	80.2	95.0	97.5	82.7	80.1	75.9	74.8	77.5	65.0	75.9	74.8	97.0	91.5	72.5	80.0	97.1	91.5
8	97.3	67.1	0	51.7	100	67.5	52.9	63.4	61.4	45.6	52.6	63.9	76.1	77.6	54.4	66.7	76.5	77.9	59.9	62.0	62.3	64.0	59.8	61.9	24.9	78.6	85.9	50.0	23.2	79
9	99.6	99.6	0	0	100	100	70.9	1.2	52.9	100	71.0	0.8	98.6	97.4	94.1	97.1	98.6	97.4	77.3	92.0	73.5	67.6	77.3	92.1	98.9	98.5	17.6	50.0	99.2	98.7
10	0.4	67.2	100	78.2	0	67.2	11.3	38.9	89.1	74.5	10.9	38.7	93.8	84.6	49.1	56.4	94.0	84.7	55.2	54.9	60.0	63.6	55.1	54.9	81.4	68.8	45.4	69.1	81.6	68.8
11	27.7	23.1	99.0	98.0	24.5	19.7	78.3	70.6	37.0	50.0	80.2	71.5	81.2	83.7	27.0	25.0	83.6	86.3	67.3	75.1	52.0	66.0	68.0	75.5	59.1	83.9	60.0	64.0	59.0	84.8
12	1.4	1.4	100	100	0	0	58.5	47.1	47.9	64.2	58.7	46.8	76.9	36.5	73.9	91.9	76.9	35.7	63.2	60.0	58.5	68.3	63.3	59.9	13.5	36.8	97.6	77.2	12.3	36.2
13	70.6	70.1	63.6	61.8	70.7	70.1	16.5	35.6	98.2	90.9	15.8	35.1	24.0	23.7	98.2	100	23.4	23.0	36.4	46.6	87.3	80.0	35.9	46.3	18.7	32.9	96.4	89.1	18.0	32.4
14	16.7	7.1	100	100	16.4	6.8	93.7	69.8	30.0	65.0	93.9	69.9	81.0	74.4	100	80.0	80.9	74.4	74.3	82.0	55.0	55.0	74.4	82.1	95.4	92.1	25.0	25.0	95.6	92.3
15	97.9	2.0	0	100	100	0	69.9	62.0	42.7	49.2	70.5	62.3	87.7	60.7	96.4	73.4	87.6	60.5	70.2	38.3	79.4	81.0	70.0	37.4	84.5	59.8	32.7	66.5	85.6	59.7
16	15.3	4.6	100	100	15.2	4.4	82.1	7419	75.0	62.5	82.1	74.1	65.5	56.9	100	100	65.5	56.8	38.5	40.9	100	87.5	38.4	40.9	80.0	74.7	75.0	75.0	80.0	74.7
17	98.6	98.5	0	0	100	99.9	62.5	66.1	61.1	52.8	62.5	66.3	85.3	84.3	63.9	77.8	85.6	84.4	70.2	63.2	83.3	80.6	70.0	62.9	87.3	74.8	27.8	50.0	88.1	75.1
18	64.3	3.6	92.3	100	64.1	2.9	80.9	84.0	51.3	53.8	81.2	84.2	84.6	79.4	82.0	82.1	84.6	79.5	65.2	55.7	87.2	82.0	65.1	55.5	74.2	80.6	61.5	74.3	74.3	80.6
19	37.8	9.7	100	100	37.1	8.7	98.1	96.6	41.4	62.1	98.7	97.0	89.7	83.3	96.5	96.5	89.6	83.1	81.4	87.9	82.7	75.9	81.3	88.1	98.8	95.3	72.4	79.3	99.2	95.4
20	53.2	55.5	100	100	52.8	55.2	99.1	98.4	33.3	44.4	99.6	98.8	99.2	98.1	75.0	77.8	99.3	98.3	94.1	89.4	66.7	80.6	94.3	89.5	99.2	98.8	16.7	55.6	99.8	99.1
21	92.2	1.1	8.3	100	92.8	0.4	44.1	56.9	75.0	66.7	43.9	56.8	89.9	80.3	91.7	87.5	89.9	80.2	67.6	77.4	62.5	62.5	67.7	77.5	63.4	24.4	58.3	95.8	63.5	23.9
22	99.0	99.0	0	0	99.9	100	51.3	55.8	56.0	56.0	51.3	55.8	89.2	88.3	92.0	92.0	89.2	88.2	39.7	51.6	76.0	88.0	39.3	51.2	4.8	39.7	84.0	44.0	4.0	39.7
23	7.6	4.4	100	100	7.0	3.8	95.6	95.8	34.6	38.5	95.9	96.2	81.9	86.2	88.5	86.5	81.9	86.2	73.3	63.3	73.1	82.7	73.3	63.1	96.6	94.1	30.8	73.1	97.1	94.2
Mean	54.9	35.5	58.8	81.3	55.1	35.0	70.7	67.6	54.2	58.8	70.9	67.8	78.4	73.3	82.0	82.1	78.5	73.3	64.6	65.9	74.3	75.9	64.6	65.8	71.5	72.2	55.7	66.8	71.6	72.3
std	40.1	34.9	48.1	34.8	40.9	35.5	24.2	24.1	17.6	16.1	24.5	24.3	19.8	21.5	21.3	20.0	20.0	21.7	15.1	15.6	13.5	10.3	15.3	15.8	32.2	24.6	26.1	17.6	32.7	24.9

Table S3. Patient-specific classification results using Feature 3 in terms of performance measures of accuracy (Acc.), sensitivity (Sens.) and specificity (Spec.), obtained using EMD and DWT-based dictionaries and 5 classifiers.

Pat. No	SVM						LDA						NB						CT						k-NN					
	Acc.		Sens.		Spec.		Acc.		Sens.		Spec.		Acc.		Sens.		Spec.		Acc.		Sens.		Spec.		Acc.		Sens.		Spec.	
	EMD	DWT	EMD	DWT	EMD	DWT	EMD	DWT	EMD	DWT	EMD	DWT	EMD	DWT	EMD	DWT	EMD	DWT	EMD	DWT	EMD	DWT	EMD	DWT	EMD	DWT	EMD	DWT	EMD	DWT
1	94.6	94.1	90.9	89.1	94.6	94.1	96.7	96.8	94.5	74.5	96.7	97.0	82.5	85.1	98.2	96.4	82.4	85.1	95.1	80.2	94.5	100	95.1	80.0	95.1	81.0	94.5	94.5	95.1	80.8
2	47.4	89.6	100	76.2	46.9	89.7	48.8	47.9	76.2	76.2	48.4	47.6	94.5	85.9	71.4	85.7	94.7	85.9	92.6	84.1	85.7	100	92.7	83.9	93.1	88.9	80.9	80.9	93.3	89.0
3	87.9	80.9	100	100	87.8	80.8	88.7	87.2	100	88.0	88.6	87.2	81.6	83.4	100	86.0	81.4	83.4	86.1	85.4	100	98.0	85.9	85.3	86.1	85.4	100	98.0	85.9	85.3
4	90.8	86.7	34.8	52.2	91.1	86.9	19.7	19.6	52.2	54.3	19.6	19.5	34.0	34.0	47.8	47.8	34.0	33.9	63.2	90.3	50.0	8.7	63.3	90.7	84.3	75.4	47.8	50.0	84.5	75.6
5	91.9	86.7	97.1	85.7	91.8	86.7	98.5	94.2	71.4	62.8	98.9	94.7	90.5	94.8	97.1	61.4	90.4	95.3	94.7	77.6	97.1	90.0	94.7	77.4	94.7	76.7	97.1	95.7	94.7	76.4
6	86.3	86.3	94.1	94.1	86.2	86.3	84.2	87.0	94.1	94.1	84.2	87.0	49.5	84.4	100	94.1	49.4	84.4	30.2	55.9	88.2	94.1	30.2	55.9	51.4	54.4	94.1	94.1	51.3	54.4
7	98.3	84.5	87.5	77.5	98.4	84.5	99.2	92.0	82.5	70.0	99.3	92.1	98.5	69.2	87.5	92.5	98.6	69.1	92.2	50.1	100	95.0	92.2	49.9	87.2	54.9	100	95.0	87.2	54.7
8	80.6	67.4	99.1	76.3	80.1	67.2	92.4	81.7	82.4	63.1	92.7	82.2	92.1	86.9	85.1	53.5	92.3	87.9	71.1	50.4	96.5	80.7	70.4	49.5	85.0	56.2	90.3	72.8	84.9	55.7
9	98.2	97.9	88.2	88.2	98.2	97.9	99.4	99.3	82.3	82.3	99.5	99.4	26.0	21.2	94.1	97.0	25.7	20.9	89.9	89.7	94.1	94.1	89.9	89.7	89.5	89.1	94.1	94.1	89.5	89.1
10	88.4	98.8	94.5	81.8	88.4	98.9	99.1	99.3	76.4	74.5	99.2	99.4	83.5	81.9	96.4	90.9	83.4	81.9	96.4	90.3	80.0	83.6	96.5	90.3	95.3	95.2	85.4	81.8	95.4	95.3
11	98.7	77.9	93.0	53.0	99.0	79.0	98.6	85.8	81.0	12.0	99.4	89.1	98.0	87.1	95.0	10.0	98.1	90.6	98.9	73.3	95.0	64.0	99.0	73.8	98.9	77.9	94.0	55.0	99.1	79.0
12	60.8	77.8	81.3	69.1	60.5	77.9	77.6	75.1	53.6	65.8	77.9	75.3	81.7	74.2	46.3	70.7	82.3	74.3	65.9	43.0	65.0	70.7	65.9	42.6	61.2	52.6	61.8	64.2	61.2	52.4
13	78.3	76.7	87.3	89.1	78.2	76.6	84.0	83.3	74.5	76.4	84.1	83.4	82.3	80.5	78.2	81.8	82.4	80.5	68.0	65.9	85.4	90.9	67.8	65.7	69.7	69.9	83.6	90.9	69.6	69.7
14	85.9	84.1	90.0	85.0	85.9	84.1	93.6	92.6	35.0	35.0	93.8	92.8	76.9	69.0	100	100	76.8	68.9	86.8	77.9	85.0	95.0	86.8	77.8	86.8	77.9	85.0	95.0	86.8	77.8
15	82.0	64.3	93.1	64.1	81.8	64.3	84.9	65.1	87.5	63.7	84.8	65.2	82.7	75.6	91.5	49.6	82.5	76.1	64.9	51.1	93.1	69.3	64.3	50.7	65.9	48.6	93.9	70.2	65.3	48.2
16	93.8	94.9	87.5	75.0	93.8	95.0	68.9	66.0	100	100	68.8	65.9	81.5	70.0	100	100	81.5	69.9	80.8	81.7	100	100	80.7	81.7	70.9	72.7	100	100	70.9	72.6
17	78.8	76.7	94.4	97.2	78.6	76.4	87.0	86.5	94.4	94.4	86.9	86.4	86.4	85.6	94.4	94.4	86.3	85.4	74.2	79.3	97.2	97.2	73.9	79.0	79.9	79.5	88.9	94.4	79.8	79.2
18	83.8	69.8	97.4	97.4	83.6	69.6	86.1	78.8	97.4	92.3	86.0	78.7	79.7	64.7	94.9	100	79.6	64.4	80.6	39.2	100	100	80.4	38.8	77.7	58.8	100	97.4	77.6	58.5
19	95.6	95.1	86.2	93.1	95.7	95.1	97.8	97.8	86.2	82.7	97.9	98.0	74.0	69.1	100	100	73.7	68.7	79.9	85.8	96.5	96.5	79.7	85.7	79.9	76.9	96.5	96.5	79.7	76.6
20	97.8	88.9	94.4	94.4	97.9	88.9	98.9	95.2	69.4	83.3	99.1	95.3	98.9	95.5	69.4	83.3	99.1	95.6	97.9	86.4	83.3	86.1	98.0	86.4	96.7	86.0	86.1	83.3	96.8	86.0
21	81.0	91.5	83.3	79.2	81.0	91.6	88.8	94.1	75.0	75.0	88.9	94.2	88.5	75.0	75.0	79.2	88.6	75.0	79.9	86.5	87.5	87.5	79.9	86.5	83.0	83.2	79.2	87.5	83.1	83.2
22	97.7	96.8	96.0	96.0	97.7	96.8	98.1	97.9	96.0	96.0	98.1	98.0	96.2	95.1	96.0	96.0	96.3	95.1	95.1	96.4	100	96.0	95.0	96.4	95.1	96.4	100	96.0	95.0	96.4
23	90.3	89.5	98.1	88.5	90.3	89.6	95.2	94.1	92.3	76.9	95.2	94.2	74.3	66.6	98.1	100	74.1	66.4	90.6	86.3	98.1	90.4	90.6	86.2	90.6	86.3	98.1	90.4	90.6	86.2
Mean	86.4	85.1	89.9	82.7	86.4	85.1	86.4	83.4	80.6	73.6	86.4	83.6	79.7	75.4	87.7	81.3	79.7	75.6	81.5	74.2	90.1	86.4	81.4	74.1	83.4	75.0	89.2	86.0	83.4	74.9
std	12.3	9.8	13.1	13.4	12.5	9.8	18.7	18.9	16.5	20.2	18.9	19.0	19.0	17.8	15.9	22.9	19.1	18.0	15.9	17.2	12.2	19.8	16.0	17.3	12.4	14.2	12.8	14.2	12.5	14.3

Table S4. Number of four-second segments per patient for dictionary creation and learning, validation, classifier training and testing seizure detection performance. The number of seizure and non-seizure segments for the first three tasks are the same, but different for testing seizure detection performance, and hence are mentioned separately. Furthermore, as mentioned in Section 2.4, the segments of all 23 EEG channels are combined for dictionary creation and learning and validation, whereas classifier training and testing tasks are performed per channel.

Number of segments					
Patient No.	Dictionary Creation and Learning	Dictionary Validation	Classifier Training	Testing (seizure)	Testing (non-seizure)
1	368	138	33	55	5815
2	138	69	13	21	1974
5	345	115	30	50	6149
4	322	115	29	46	9447
5	483	161	42	70	4289
6	115	46	11	17	23250
7	276	115	24	40	8011
8	782	276	69	114	4155
9	230	92	21	34	8250
10	391	138	33	55	12448
11	690	253	60	100	2212
12	851	299	74	123	8343
13	368	138	33	55	6134
14	138	69	13	20	6235
15	1725	575	150	248	11860
16	69	23	5	8	4473
17	253	92	22	36	2595
18	276	92	24	39	4949
19	207	69	18	29	2547
20	253	92	22	36	4898
21	161	69	15	24	3372
22	184	69	15	25	2622
23	368	138	32	52	7903
